# Immunomodulatory effects of *Sanghuangporus sanghuang* polysaccharide SSP1 in cyclophosphamide-induced immunosuppressed mice: associations with gut microbiota, short-chain fatty acids, and metabolic remodeling

**DOI:** 10.3389/fimmu.2026.1931873

**Published:** 2026-09-17

**Authors:** Kun Liu, Xinyao Fu, Yihang Liu, Chongpeng Zhong, Zejuan Kong, Qingsheng Zhao, Junpeng Wang, Yu Xu, Hua Cheng

**Affiliations:** 1 College of Bioscience and Bioengineering, Hebei University of Economics and Business, Shijiazhuang, China; 2 College of Statistics & Mathematics, Hebei University of Economics and Business, Shijiazhuang, China; 3 Lee Kong Chian School of Medicine, Nanyang Technological University, Singapore, Singapore; 4 Institute of Biology, Hebei Academy of Sciences, Shijiazhuang, China; 5 State Key Laboratory of Biopharmaceutical Preparation and Delivery, Institute of Process Engineering, Chinese Academy of Sciences, Beijing, China; 6 Institute of Infection and Immunity of Huaihe Hospital, Henan University, Kaifeng, China; 7 Department of Neurology, The 980th Hospital of the Joint Logistic Support Force of the PLA, Shijiazhuang, China

**Keywords:** gut microbiota, immunomodulatory, polysaccharide, *Sanghuangporus sanghuang*, short-chain fatty acids

## Abstract

**Introduction:**

Immunosuppression induced by chemotherapeutic agents such as cyclophosphamide (CTX) is frequently associated with gut microbiota dysbiosis. *Sanghuangporus sanghuang* polysaccharide (SSP1) possesses immunomodulatory potential; however, its effects on mucosal immunity and the gut microbiota remain unclear.

**Methods:**

In this study, we investigated the immunomodulatory activity of SSP1 in CTX-induced immunosuppressed mice. Mice were treated with different doses of SSP1 orally. We assessed immune organ indices, histopathology of immune organs and colon, oxidative stress, splenocyte proliferation, T-cell subsets, and cytokine production. Additionally, 16S rRNA sequencing was used to analyze gut microbiota composition; targeted analysis was performed on short-chain fatty acids (SCFAs); and untargeted metabolomics was used to evaluate metabolic profiles.

**Results:**

Oral administration of SSP1 significantly alleviated immunosuppression in a dose-dependent manner, with the high dose (SSP1-H) showing the most pronounced effects. This was evidenced by restored body weight, increased spleen and thymus indices, improved histopathology, and attenuated oxidative stress. SSP1 promoted splenocyte proliferation, elevated CD45^+^ leukocytes and CD3^+^ T cells, enhanced IL-2 and IL-4 production, suppressed IFN-γ secretion, and inhibited LPS-induced pro-inflammatory cytokines (IL-1β, IL-6, TNF-α). Gut microbiota analysis revealed that SSP1-H increased α-diversity, enriched beneficial bacteria (*Bacteroides* and *Odoribacter*), and elevated SCFAs (acetate, propionate, and valerate). Correlation analysis showed SCFA levels were positively correlated with antioxidant markers, T-cell subsets, and IL-10, and negatively correlated with pro-inflammatory cytokines. Untargeted metabolomics further indicated that SSP1-H regulated serine, threonine, and taurine metabolism.

**Discussion:**

Collectively, these findings suggest that high-dose SSP1 treatment was associated with coordinated changes in gut microbiota composition, SCFA production, and immune parameters, supporting a potential microbiota–metabolism–immune axis. This implies that SSP1’s protection against CTX-induced immunosuppression is associated with regulating immune cell functions and reshaping the gut microbiota to enhance SCFA production, providing new insights into the immunoregulatory mechanisms of Sanghuang polysaccharides and highlighting their potential as functional food ingredients or immune adjuvants.

## 1 Introduction

Cyclophosphamide (CTX) is a widely used agent and is commonly used to establish experimental models of immunosuppression. It exerts broad immunosuppressive effects by inhibiting the activity of bone marrow hematopoiesis, blocking lymphocyte proliferation and differentiation, and reducing the secretion of key immune-related cytokines, thereby impairing both cellular and humoral immune functions (1, 2). Notably, CTX-induced immunosuppression is, in most cases, coupled with severe gut microbiota dysbiosis, which not only compromises the intestinal barrier but may also exacerbate systemic immune dysfunction via the gut-immune axis (3, 4).

Over the past decades, natural products have gained growing interest as immunomodulatory candidates by virtue of their multi-targeting properties and relatively low toxicity (5, 6). Within these natural components, polysaccharides are categorized as a vital subset of bioactive macromolecules that have been extensively demonstrated to possess significant immunoenhancing and immunoregulatory activities. These effects are mediated through multiple mechanisms, including activation of macrophages, promotion of lymphocyte proliferation and differentiation, and modulation of cytokine secretion (7, 8). Accumulating evidence suggests that many polysaccharides are able to withstand digestive breakdown in the upper gastrointestinal tract and can reach the colon, where they are utilized by commensal microorganisms (9). On the one hand, polysaccharides can serve as prebiotics, preferentially promoting the proliferation of probiotic microbes (e.g., *Lactobacillus*) while suppressing the proliferation of harmful pathogens, thereby improving the intestinal microbial homeostasis (10). On the other hand, their microbial fermentation products, particularly short-chain fatty acids (SCFAs), are capable of exerting direct effects upon intestinal immune cells, reinforcing barrier function, and regulating systemic immune responses, constituting a critical pathway for microbiota-immune regulation (1, 11, 12).

*Sanghuangporus* spp. are rare edible and medicinal fungi whose therapeutic value was first documented in *Shennong’s Classic of Materia Medica*. They are historically applied as folk remedies against multiple health complaints, including diarrhea, menstrual disorders, and stomachache (13, 14). Polysaccharides are counted among the principal pharmacologically active ingredients of *Sanghuangporus* spp. and are characterized by considerable structural complexity. So far, in excess of 60 polysaccharide constituents have been successfully purified from *Sanghuangporus* spp., although just a minority of these candidates have undergone detailed structural characterization (13). Earlier investigations have confirmed that the polysaccharides are primarily composed of mannose, glucose, and galactose with different molar percentages, plus supplementary monosaccharides like rhamnose and arabinose also detected (15, 16). Functionally, *Sanghuangporus* polysaccharides possess an extensive array of bioactive properties, covering anti-tumor, antioxidant, anti-inflammatory, and immunomodulatory effects (13). In particular, their immunomodulatory properties have garnered increasing attention. These polysaccharides have been reported to activate macrophages, promote lymphocyte proliferation, modulate the production of immune cytokines (for instance, TNF-α, IFN-γ, IL-2), and enhance natural killer cell activity, thereby exerting multiple-level immune regulatory effects (17–20).

Despite accumulating evidence supporting the immunomodulatory activity of *Sanghuangporus sanghuang* polysaccharides (SSP), existing studies remain largely confined to *in vitro* experiments (21, 22). Consequently, whether SSP can effectively alleviate chemotherapy-induced immunosuppression *in vivo*, and whether such effects exert impacts via reshaping the structural configuration of enteric microbes and function, remain unresolved. Given the well-established regulatory impact of enteric flora alongside derived metabolites in regulating the development of host immune responses, it is reasonable to speculate that the immunoprotective effects of SSP may extend beyond direct immune cell activation and involve microbiota-dependent mechanisms.

Accordingly, we hypothesized that SSP administration could attenuate CTX-induced immunosuppression by restoring gut microbial homeostasis, enhancing the production of beneficial microbial metabolites (particularly SCFAs), and reshaping host metabolic pathways associated with immune regulation and oxidative stress. To investigate this hypothesis, a CTX-triggered immunosuppressed mice model was utilized to systematically assess the impact of SSP on immune organ indices, lymphocyte functionality, and circulating markers of inflammation and oxidative stress. Moreover, an integrative multi-omics approach, incorporating 16S rRNA gene sequencing, targeted SCFA analysis, and untargeted metabolomics, was implemented to thoroughly characterize SSP-induced changes in gut microbiota composition, microbial metabolic activity, and host systemic metabolism. Consequently, this research seeks to characterize the association between SSP1-induced microbiota remodeling, microbial metabolite production, and immune restoration, thereby providing experimental evidence for a potential microbiota–metabolism–immune regulatory axis.

## 2 Materials and methods

### 2.1 Sample fabrication and content quantification for *S. sanghuang* polysaccharide

The fruiting bodies of *S. sanghuang* were pulverized, steeped within 95% ethanol during 24 h, filtered, dried, and sieved. Ultrapure water was added to preprocessed powder at a solid-liquid ratio of 1:110 (g/mL), then the mixture was kept at 100 °C for 10 h to complete aqueous extraction. After filtering and concentrating the extract, freeze-drying was performed to acquire crude *S. sanghuang* polysaccharide.

Pigments as well as proteins existing in crude polysaccharide were removed using polyamide chromatography, followed by further purification by a DEAE-52 ion exchange column, as previously described (23). The resulting fraction was designated SSP1. The total sugar content of SSP1 was determined by the phenol-sulfuric acid method.

### 2.2 Component composition analysis of SSP1

SSP1 (20 mg) was hydrolyzed in 2 mol/L trifluoroacetic acid (TFA) within sealed tubes under 121 °C for 2 h. Post-hydrolysis treatment applied three rounds of methanol azeotropic distillation to strip off residual TFA. The dried hydrolysate was dissolved in ultrapure water, evaporated under nitrogen, and transferred to chromatographic vials for analysis.

Referring to the published experimental scheme from (24), chromatographic measurement was conducted on Thermo ICS500 ion chromatography equipment (Thermo Fisher Scientific, USA), which was outfitted with electrochemical detector alongside Dionex™ CarboPac™ PA20 column (150 mm × 3.0 mm, 10 μm, Thermo Fisher, America). The chromatographic column was thermostatted at 30 °C; sample injection volume was 5 μL and the mobile phase pumped at 0.5 mL/min.

The mobile phase consisted of Phase A (water), Phase B (0.1 mol/L NaOH), and Phase C (0.1 mol/L NaOH + 0.2 mol/L NaAc). The elution program was as follows: 0–26 min, A:B: C = 95:5:0 (v/v/v); 26–42 min, A:B: C = 85:5:10; 42–42.1 min, A:B: C = 60:0:40; 42.1–52 min, A:B: C = 60:40:0; 52.1–60 min, A:B: C = 95:5:0.

Identification of monosaccharide components was realized by aligning their chromatographic retention durations with certified standard monosaccharides (Sigma-Aldrich, USA), including ribose, fructose, mannose, xylose, glucose, galactose, rhamnose, arabinose, fucose, mannuronic acid, glucuronic acid, guluronic acid, and galacturonic acid. The molar ratios were calculated based on standard calibration curves.

### 2.3 Animals and experimental design

Female Balb/c mice at six weeks of age were sourced from the Laboratory Animal Facility of Hebei Medical University. All animal manipulations complied with NIH-released guidelines regulating laboratory animal welfare and experimental handling. Mouse specimens were maintained within ventilated isolated cage racks at constant environmental parameters (24 ± 3 °C, alternating 12 h light and dark), allowing ad libitum intake of feed and sterile water.

After one week of acclimatization (**Figure 1A**), mice were randomly divided into six groups (12 mice/group): normal control, model, positive control (levamisole hydrochloride, 40 mg/kg/day), SSP1 low-dose (100 mg/kg/day), SSP1 medium-dose (200 mg/kg/day), and SSP1 high-dose (400 mg/kg/day). Doses were selected based on previous studies (25).

**Figure 1:**
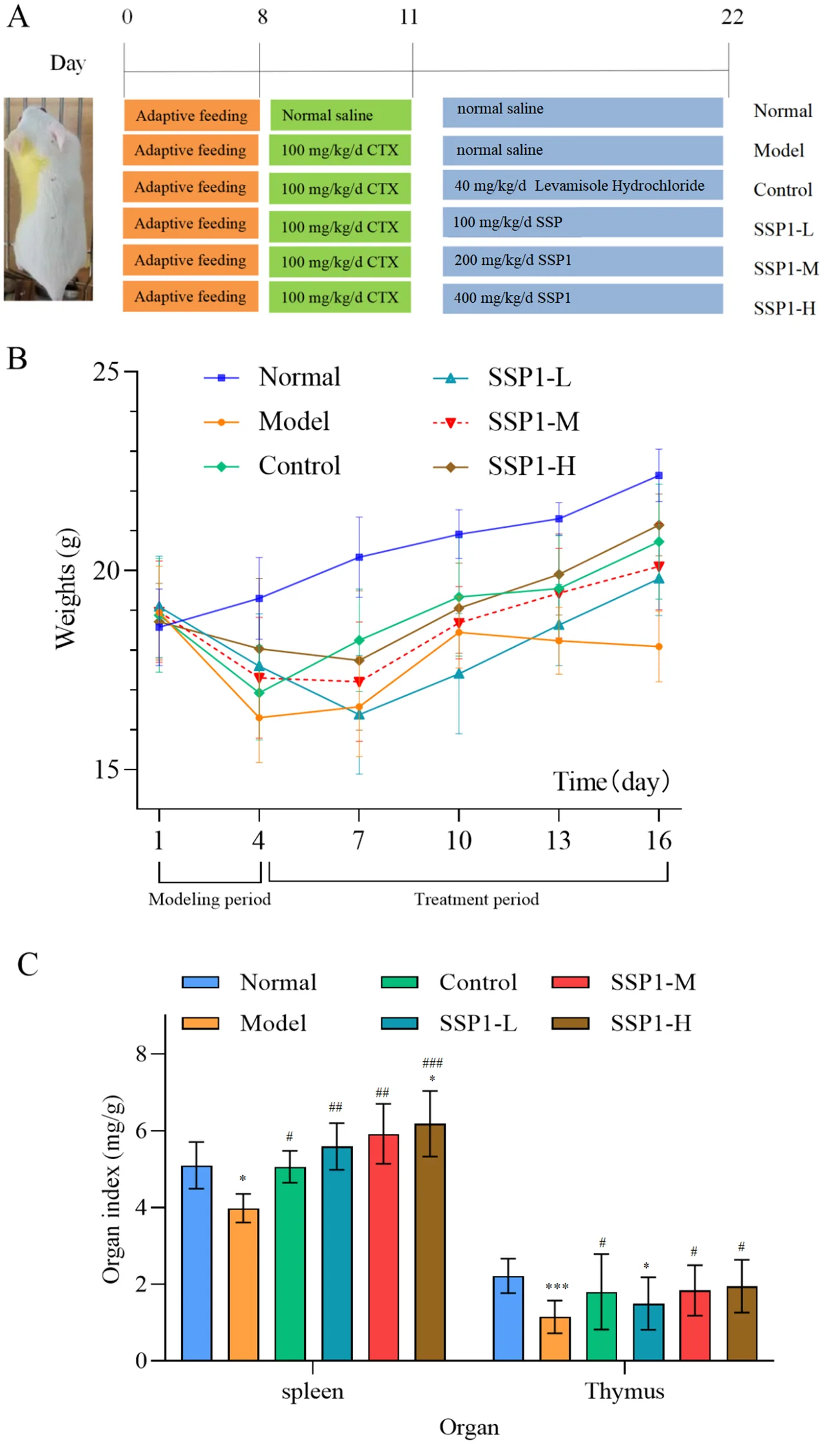
Experimental study design **(A)**, and the effects of SSP1 on body weight **(B)** and organ index **(C)** in CTX-induced mice. (*P < 0.05, **P < 0.01 and ***P < 0.001 vs. the Normal group; ^#^*P* < 0.05, ^##^*P* < 0.01 and ^###^*P* < 0.001 vs. the Model group; Mean ± SD, n=10).

With the healthy control cohort excluded, every experimental mouse was subjected to intraperitoneal injection treatment of CTX (purity > 99%; Beyotime, Shanghai, China) at 100 mg/kg once daily for three consecutive days (days 8, 9, and 10). From day 11 onward, mice in the treatment groups were administered the corresponding agents via daily gavage lasting 12 consecutive days. Control groups received an equal volume of sterile saline. Body weight was recorded daily. All animal experimental procedures were approved by the Animal Experimental Ethics Committee of the Institute of Biology, Hebei Academy of Sciences (Approval No.: SWS01120).

A total of 72 mice were initially enrolled (12 per group). After accidental deaths during gavage, the final cohort was adjusted to 10 mice per group for body weight, organ index, and histopathological evaluations (5 mice per group for histology). Due to limited serum and fecal volumes, random blinded subsets were assigned: 5 mice per group for cytokine/oxidative stress assays; 3 mice per group for flow cytometry and splenocyte functional assays; and 5 mice (16S sequencing) or 3 mice (SCFA quantification) for microbiota analyses, based on sample availability.

### 2.4 Sample collection and immune organ index

On day 23, mice were euthanized via cervical dislocation. Serum, thymus, spleen, feces, colon, and luminal colonic digesta were collected for subsequent analyses. Calculation for immune organ coefficient relied on dividing each isolated organ’s weight (mg) by the whole-body weight (g) of the tested animal before euthanasia.

### 2.5 Serum cytokines and antioxidant indices

Serum levels of TNF-α, IL-10 and IFN-γ were measured using their corresponding ELISA kits (all from Beyotime Biotechnology Co., Ltd., Shanghai, China). Superoxide dismutase (SOD) and catalase (CAT) activities, as well as malondialdehyde (MDA), were determined using commercial kits (Nanjing Jiancheng Bioengineering Institute, Nanjing, China), following the manufacturers’ instructions.

### 2.6 Flow cytometric analysis of immune cell populations

Splenocytes were isolated as previously described (26). Multicolor flow cytometry was performed using antibodies against CD3, CD19, F4/80, and NK1.1 (all from eBioscience, San Diego, CA). Data were acquired using an Attune™ NxT flow cytometer (Invitrogen Attune NxT, Thermo Fisher Scientific, USA) and analyzed with Attune™ Cytometric Software v6.2.3.

### 2.7 Cell proliferation

Splenocyte suspensions were seeded in 96-well round-bottom plates at a density of 3×10^6^ cells per well, and stimulated with concanavalin A (ConA, 1.5 µg/mL, Sigma-Aldrich) for 72 h. CCK-8 reagents were added 3 h before the end of stimulation, and absorbance was measured using a microplate reader (SpectraMax i3x, Molecular Devices, USA). Cell proliferation was calculated as:


$$\text{Cell proliferation}=\frac{\text{Atreated }-\text{Ablank }}{\text{Acontrol}-\text{Ablank }}\times 100$$

### 2.8 Cytokine secretion by splenocytes

After 12 days of SSP1 administration in mice, the spleens were harvested, and lymphocyte suspensions were prepared. To detect the IL-2, IFN-γ and IL-4 secreted by T cells (all purchased from BD Bioscience), Splenocytes were stimulated with ConA or precoated anti-CD3 (5 µg/ml)/soluble anti-CD28 (1 µg/ml) antibodies for 48 h to detect IFN-γ, IL-2, and IL-4 secretion using ELISA kits (all from Beyotime Biotechnology Co., Ltd., Shanghai, China). Alternatively, cell cultures received lipopolysaccharide (LPS, Sigma-Aldrich) stimulation over a 24-hour incubation to determine TNF-α, IL-1β and IL-6 levels (all Beyotime Biotechnology Co., Ltd., Shanghai, China).

### 2.9 Histological analysis

Colon, thymus, and spleen tissues were harvested and fixed in 4% paraformaldehyde for 24–48 h, embedded in paraffin, sectioned (5 μm), and stained with hematoxylin and eosin (HE). Histomorphological changes were examined using a light microscope.

### 2.10 Short-chain fatty acid analysis

SCFA contents were quantified by GC–MS as previously described (27). Briefly, samples were mixed with 20% phosphoric acid containing 4-methylvaleric acid (internal standard), vortexed, centrifuged, and the supernatant was analyzed using an Agilent DB-FFAP column and Agilent 5977B MS system operating in SIM mode.

### 2.11 Gut microbiota analysis

Fecal DNA was extracted from mouse feces, and the V3-V4 regions of bacterial 16S rRNA were amplified using the primers 338F (5′-ACTCCTACGGGAGGCAGCAG-3′) and 806R (5′-GGACTACHVGGGTWTCTAAT-3′) (28). Sequencing procedures were completed using the Illumina MiSeq PE250 sequencing platform (Shanghai Yuanshen Biomedical Technology Co., Ltd.). Operational taxonomic unit (OTU) clustering, taxonomic annotation, and diversity analyses were conducted to characterize microbial community structure.

### 2.12 Untargeted metabonomics analysis

For metabolite extraction, 0.1 g fecal specimen was treated with methyl alcohol preloaded with 2-chloro-L-phenylalanine for internal standardization. UHPLC–MS/MS analysis was conducted using a Thermo Vanquish UHPLC system coupled with a Q Exactive mass spectrometer. Metabolite annotation was performed using KEGG, HMDB, and LipidMaps databases. Multivariate and univariate analyses were performed using MetaX software.

### 2.13 Statistical analysis

All values are reported as mean ± SD. Group comparisons were conducted using one-way ANOVA with Duncan’s multiple range test, and *P* < 0.05 was taken to indicate statistically significant.

## 3 Results

### 3.1 Monosaccharide composition

The polysaccharide content of SSP1 was 70.25 ± 0.03 percent. HPLC was used to determine the retention duration and establish a standard curve for individual monosaccharide (**Figure 2A**). The results showed that SSP1 consisted of galactose (Gal), mannose (Man), fucose (Fuc), glucose (Glc), rhamnose (Rha), xylose (Xyl), arabinose (Ara), and glucuronic acid (Glc-UA), at a molar proportion of 34.24:21.30:20.81:16.95:2.83:2.50:1.04:0.33, respectively (**Figure 2B**).

**Figure 2:**
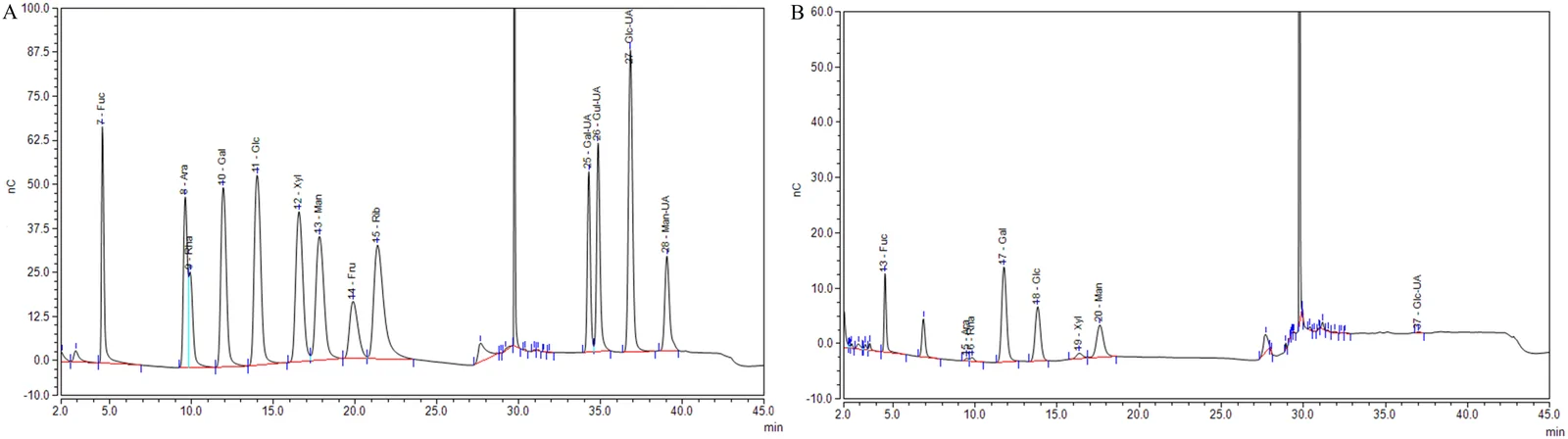
HPLC chromatograms for Standards **(A)** and SSP1 **(B)**.

### 3.2 Effects of SSP1 on body weight and immune organ indices of mice

As shown in **Figure 1B** and **Supplementary Table 1**, mice from the model group presented reduced body weight gain versus the normal group (*P* < 0.001), which verified the valid establishment of an immunodepression mouse model. Administration of high-dose SSP1 improved body weight recovery in immunosuppressed mice (*P* < 0.01 vs. model group), indicating that oral SSP1 alleviated CTX-induced weight loss.

The thymus index and spleen indices are widely used indicators of immune system status. As shown in **Figure 1C**, both indices displayed prominent reductions for the disease-model group versus normal untreated mice (*P* < 0.05), suggesting that cyclophosphamide (CTX) induced significant immunotoxicity. Treatment with medium- and high-dose SSP1 increased both the thymus index and spleen indices (*P* < 0.05) following a dose-dependent pattern. Collectively, the obtained data indicate that SSP1 administration effectively promoted the recovery of immune organs damaged by CTX treatment.

### 3.3 Effect of SSP1 on cytokine levels and oxidative stress in immunosuppressed mice

As shown in **Figures 3A–C**, serum levels of IFN-γ, IL-10, and TNF-α were significantly lower in the model group than in the normal group (P < 0.05), indicating suppressed immune response. SSP1 administration elevated the levels of these cytokines along a dosage-proportioned trend, and maximal therapeutic efficacy was observed within the high-concentration group (*P* < 0.01).

**Figure 3:**
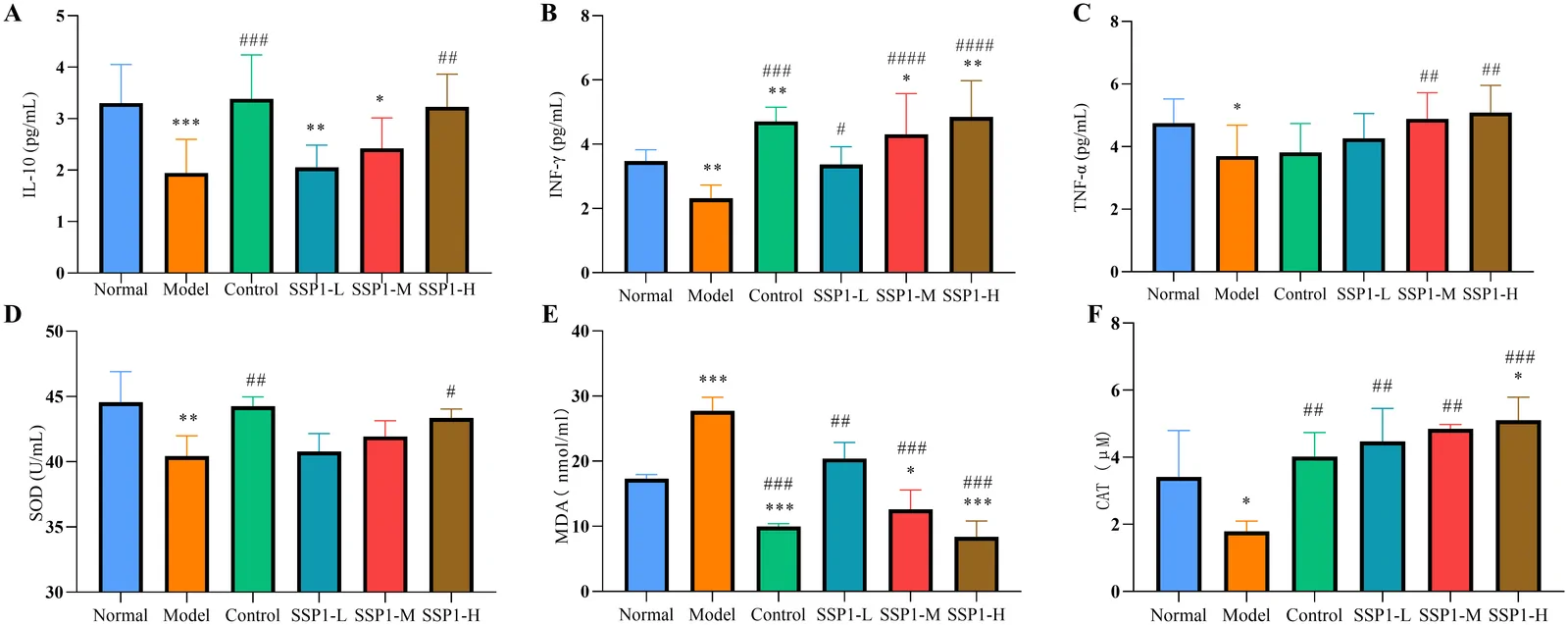
Effect of SSP1 on the levels of IL-10 **(A)**, INF-γ **(B)**, TNF-α **(C)**, SOD **(D)**, MDA **(E)**, and CAT **(F)** in mice. (*P < 0.05, **P < 0.01 and ***P < 0.001 vs. the Normal group; ^#^*P* < 0.05, ^##^*P* < 0.01 and ^###^*P* < 0.001 vs. the Model group; Mean ± SD, n=5).

CTX treatment also induced oxidative stress. Results from **Figures 3D, E** revealed obvious rises in malondialdehyde content for the immunosuppressed animals (*P* < 0.001), whereas, the functional activity of two key antioxidant enzymes (SOD and CAT) underwent notable drops (*P* < 0.05), indicating disruption of the oxidative balance. SSP1 treatment counteracted the above shifts in a dosage-proportionate fashion, suggesting that SSP1 alleviated oxidative stress in immunosuppressed mice.

### 3.4 SSP1 reduced pathological injury in the spleen and gut

With regard to colonic specimens (**Figure 4A**), animals belonging to the blank control cohort had intact colonic mucosa structures, regularly structured crypt compartments with rich goblet cell populations. In comparison, the immunosuppression group’s colon tissues showed inflammatory cell infiltration, minor crypt damage, and depletion of goblet cells. SSP1 treatment dose-dependently improved colon morphology, with reduced inflammatory infiltration and restoration of goblet cells.

**Figure 4:**
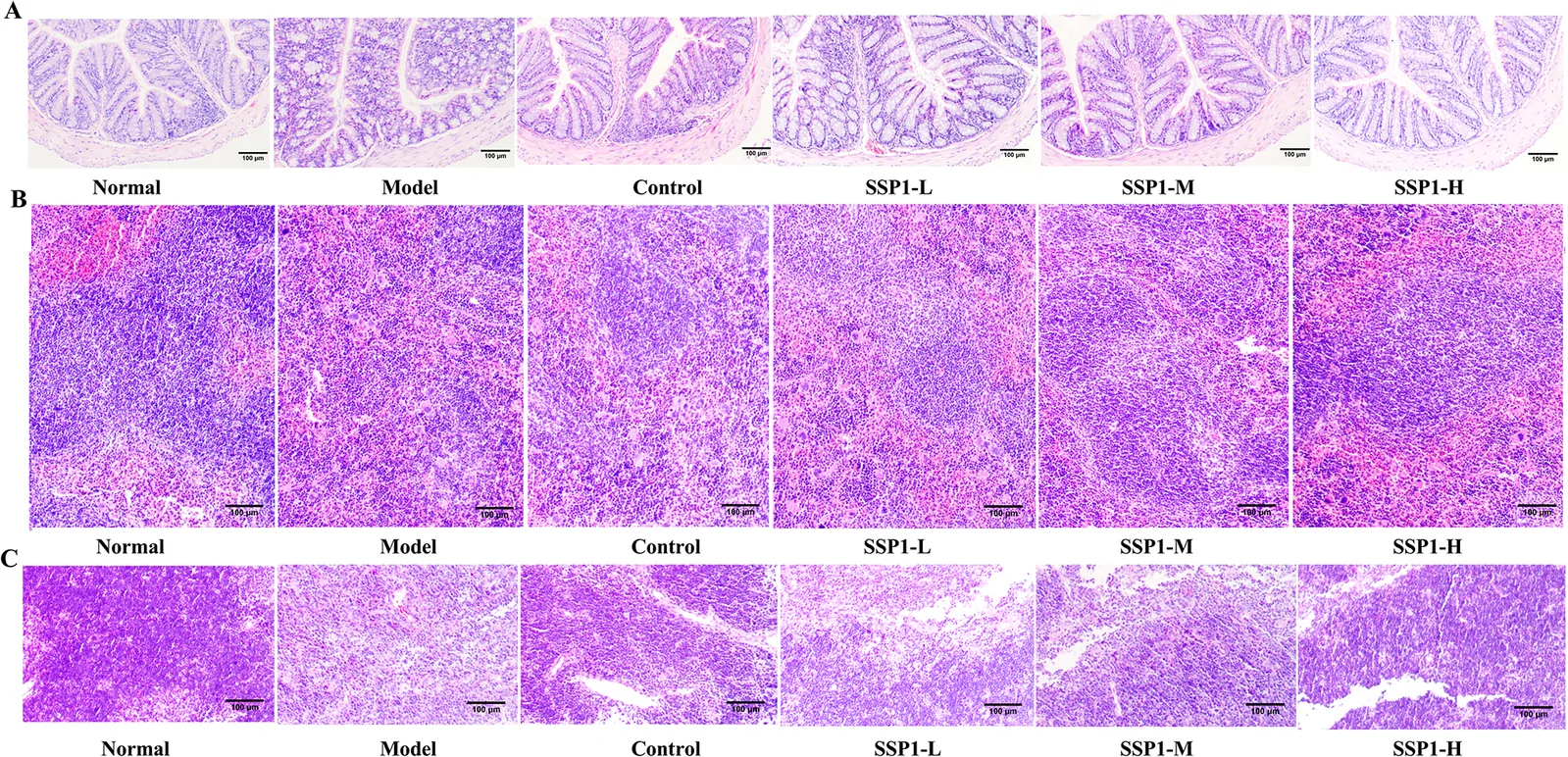
Alleviation of CTX-induced histopathological damage in colon **(A)**, spleen **(B)**, and thymus **(C)** tissues by SSP1, as shown in H&E-stained sections. (100×; scale bar = 100 μm).

In the spleen (**Figure 4B**), the normal group exhibited densely arranged splenocytes and a clear boundary between the red and white pulp. In the model group, splenocytes appeared sparse and disorganized, with nuclear condensation and blurred red–white pulp boundaries. SSP1 treatment gradually alleviated these pathological changes. In particular, the SSP1-H group showed densely arranged splenocytes and a well-defined red–white pulp structure similar to that of the normal group.

In the thymus (**Figure 4C**), the normal group showed a clear cortex–medulla boundary with densely packed lymphocytes. In contrast, the model group displayed structural loosening, blurred corticomedullary boundaries, enlarged intercellular spaces, and reduced lymphocyte numbers. SSP1 treatment progressively restored thymic architecture. In the SSP1-M and SSP1-H groups, the thymic morphology closely resembled that of the normal group, with clearer boundaries and increased lymphocyte density.

### 3.5 Effects of SSP1 on splenic lymphocyte proliferation

As shown in **Figure 5A**, SSP1 administration enhanced ConA-induced splenocyte proliferation at doses of 100, 200, and 400 mg/kg. SSP1-H showed a numerically higher proliferation response than the positive control, although the difference was not statistically significant. Nevertheless, these results indicate that SSP1 effectively promoted lymphocyte activation, achieving comparable efficacy to the positive control drug.

**Figure 5:**
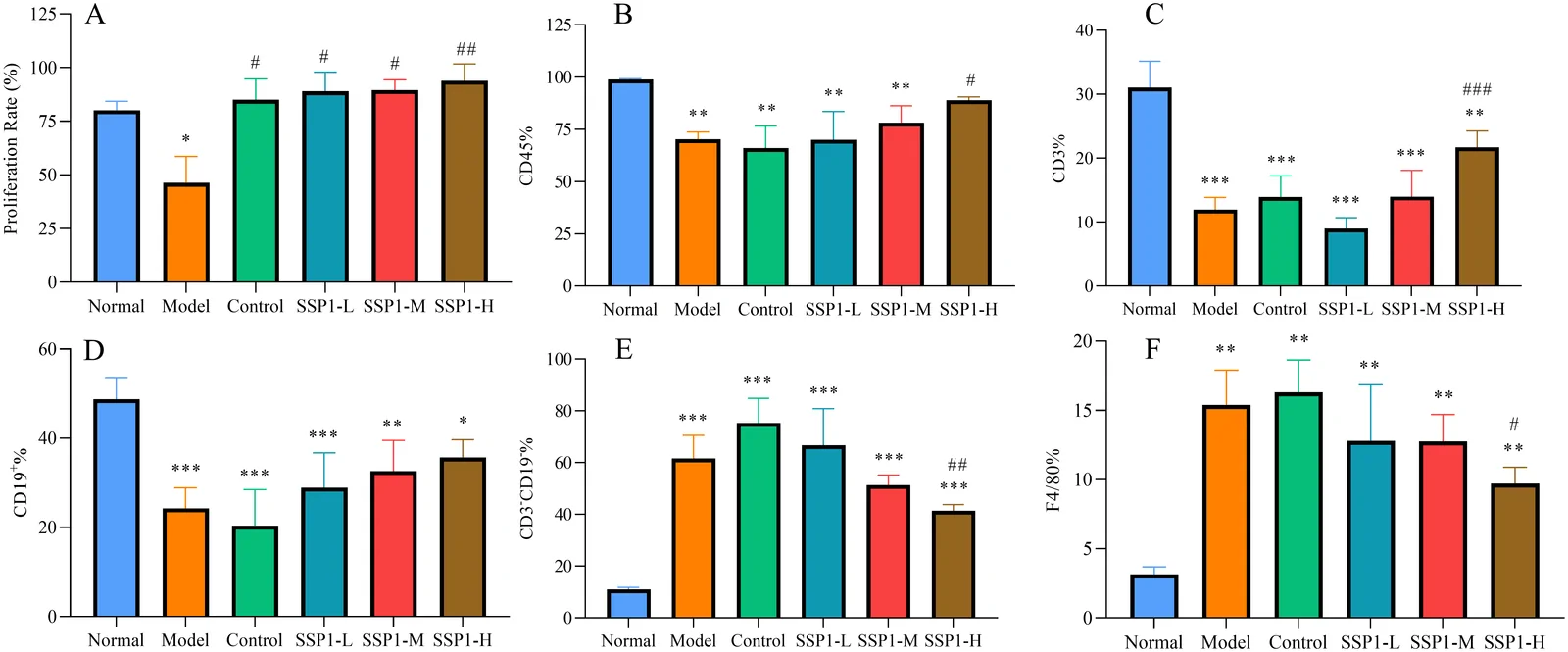
Effects of SSP1 treatment on splenic lymphocyte proliferation **(A)** and immune cell subsets **(B–F)** in mice. On day 22, spleens were collected from mice in the different-dose SSP1 treatment groups to identify immune cell subsets, including T cells (CD3^+^; **C**), B lymphocytes (CD19^+^; **D**), NK cells (CD3^-^CD19^-^; **E**), and macrophages (F4/80^+^; **F**). (*P < 0.05, **P < 0.01 and ***P < 0.001 vs. the Normal group; ^#^*P* < 0.05, ^##^*P* < 0.01 and ^###^*P* < 0.001 vs. the Model group; Mean ± SD, n=3).

### 3.6 Effects of SSP1 on splenic immune cell subsets

Flow cytometry analysis revealed that SSP1 significantly modulated immune cell populations in the spleen. Compared with the model group, the SSP1-H group showed significantly increased proportions of CD45^+^ leukocytes and CD3^+^ T lymphocytes (**Figures 5B, C**).

In contrast, the proportions of NK cells (CD3^-^CD19^-^NK1.1^+^) and macrophages (F4/80^+^) were significantly reduced (**Figures 5E, F**). The proportion of B lymphocytes (CD19^+^) increased in all SSP1-treated groups but did not reach statistical significance compared with the model group (**Figure 5D**). Gating strategies for T cells, NK cells, and macrophages are shown in **Supplementary Figure 1**.

### 3.7 Effects of SSP1 on cytokine secretion by splenic T cells

Compared with the control group, oral administration of SSP1 at 200 and 400 mg/kg significantly increased the secretion of IL-2 and IL-4 by splenic T cells following stimulation with anti-CD3/CD28 antibodies (**Table 1**). However, IFN-γ production by splenocytes under T-cell mitogen stimulation was significantly lower than that in the model group and returned to near-normal levels.

**Table 1:** Effect of SSP1 on cytokine production of splenocytes lymphocytes stimulated with mitogens.

Stimulators	Cytokines	Normal	Model	Control	SSP1-L	SSP1-M	SSP1-H
CD3/CD28	IL-2(ng/mL)	0.80 ± 0.05	0.72 ± 0.06	0.76 ± 0.08	0.80 ± 0.10	1.03 ± 0.11^**#^	1.25 ± 0.13^**##^
	IL-4(ng/mL)	0.89 ± 0.10	0.83 ± 0.03	1.10 ± 0.17	0.97 ± 0.08	1.34 ± 0.38^**##^	1.44 ± 0.08^**##^
	IFN-γ (ng/mL)	0.42 ± 0.10	0.72 ± 0.24^**^	0.50 ± 0.11^#^	0.52 ± 0.04	0.52 ± 0.11^#^	0.52 ± 0.04^#^
	IFN-γ/IL-4	0.31 ± 0.18	0.86 ± 0.21^**^	0.45 ± 0.02^#^	0.54 ± 0.01	0.39 ± 0.025^#^	0.36 ± 0.01^#^
ConA	IL-2(ng/mL)	9.61 ± 6.80	3.63 ± 1.93	6.41 ± 5.01	15.28 ± 5.55#	17.17 ± 8.06^#^	16.56 ± 10.17^#^
	IL-4(ng/mL)	0.46 ± 0.11	1.15 ± 0.14^*^	1.09 ± 0.17^*^	1.16 ± 0.10^*^	1.08 ± 0.21^*^	1.02 ± 0.15^*^
	IFN-γ (ng/mL)	0.13 ± 0.03	0.37 ± 0.06^**^	0.35 ± 0.03^**^	0.27 ± 0.03^**##^	0.28 ± 0.02^**##^	0.23 ± 0.03^**##^
	IFN-γ/IL-4	0.14 ± 0.11	0.32 ± 0.01^*^	0.32 ± 0.02^*^	0.23 ± 0.01	0.26 ± 0.03	0.23 ± 0.01
LPS	IL-1β(pg/mL)	4.38 ± 0.78	7.62 ± 1.51^**^	4.01 ± 0.74^##^	3.75 ± 0.13^##^	3.91 ± 0.62^##^	4.17 ± 0.56^##^
	IL-6(pg/mL)	33.07 ± 5.81	183.68 ± 10.04^**^	157.73 ± 19.47^**^	111.82 ± 25.45^**##^	126.87 ± 9.58^**##^	138.94 ± 7.21^**##^
	TNF-α(pg/mL)	23.46 ± 11.37	156.56 ± 69.96^**^	124.22 ± 28.60^**^	103.51 ± 28.32^**#^	100.26 ± 29.60^**#^	96.20 ± 11.94^**#^

*P < 0.05 and **P < 0.01 vs. the Normal group; ^#^*P* < 0.05 and ^##^*P* < 0.01 vs. the Model group; n=5.

Similarly, under ConA stimulation, SSP1 at all tested doses significantly increased IL-2 production while reducing IFN-γ secretion. In addition, SSP1 maintained the elevated IL-4 level observed in the model group and significantly decreased the IFN-γ/IL-4 ratio, indicating a shift of the immune response toward a Th2-dominant profile.

### 3.8 Effect of SSP1 on inflammatory cytokine production in the spleen

SSP1 exhibited a clear inhibitory effect on LPS-induced inflammation. Compared against untreated healthy specimens, the model group displayed prominent elevation in serum contents of IL-6, IL-1β as well as TNF-α, confirming successful induction of systemic inflammation.

SSP1 treatment markedly reduced these inflammatory cytokines. Among them, the inhibition of TNF-α was most pronounced and showed a dose-dependent trend, with the minimum content detected within the SSP1-H group. Similarly, IL-6 levels were significantly lower in all SSP1 treatment groups compared with the model group. Interestingly, IL-1β levels in all dosing cohorts displayed lowered cytokine values that fell beneath the measurements obtained from untreated control animals (**Table 1**).

### 3.9 SSP1-H alters the gut microbial community

To explore if SSP1-H could modulate the compositional profile of gut flora, 16S rRNA gene sequencing was conducted using fecal specimens from the normal, model, and SSP1-H groups. After quality filtering, 1,354,351 reliable sequencing reads were acquired from 15 specimen replicates, corresponding to a mean value of 90,290 sequences per specimen (**Supplementary Table 2**).

Rarefaction curves reached a plateau when the number of sequences exceeded 20,000, indicating sufficient sequencing depth to capture microbial diversity (**Figure 6A**). Rank-abundance curves also showed uniform species distribution across groups, confirming the reliability of the sequencing data (**Figure 6B**).

**Figure 6:**
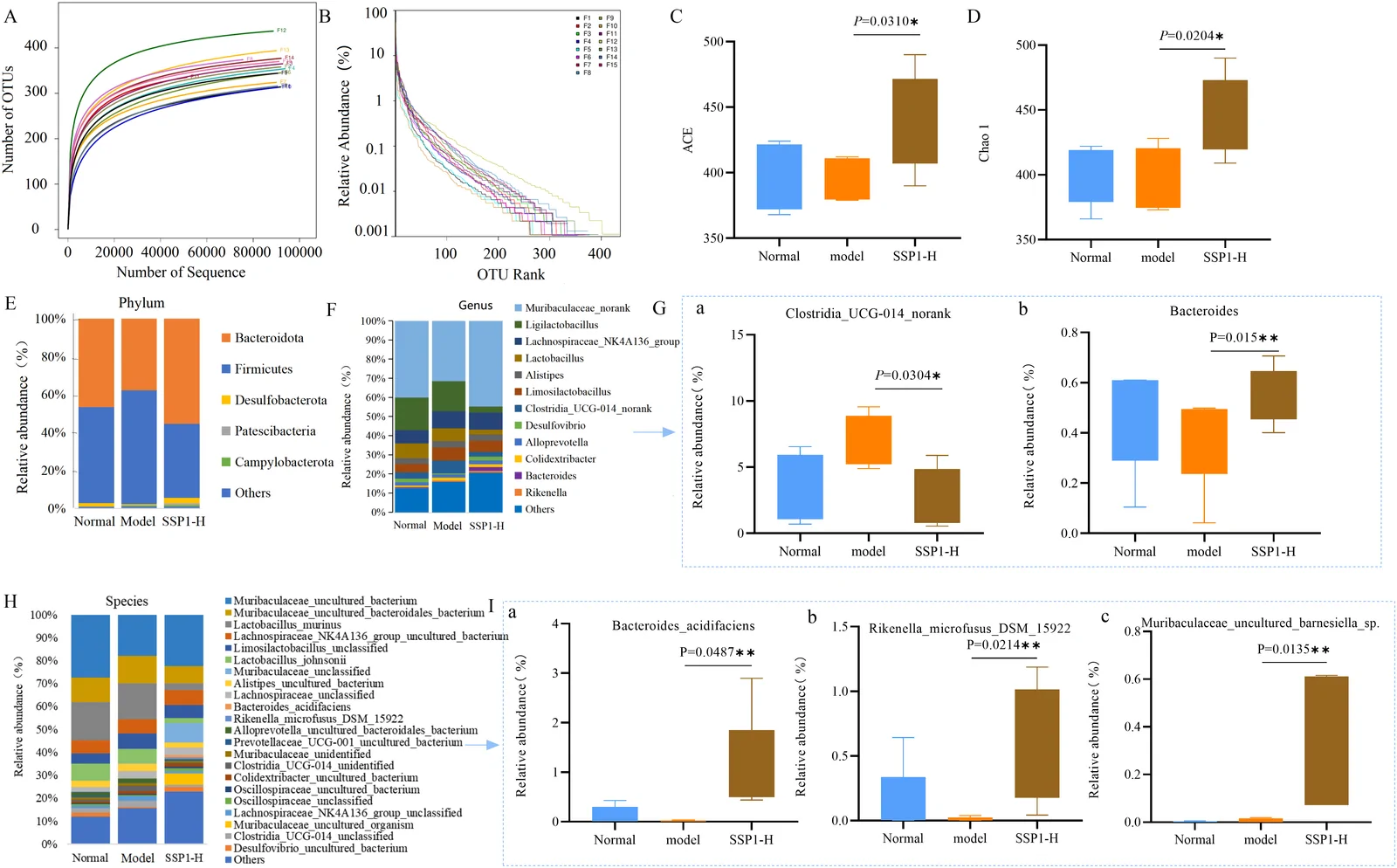
Effects of SSP1-H on gut microbiota structure in fecal. **(A)** α-diversity – ACE; **(B)** α-diversity – Chao1; **(C)** α- diversity – Shannon; **(D)** α-diversity – Simpson; **(E)** β-diversity – PCA; **(F)** microbiota composition – phylum level; **(G)** microbiota composition – genus level; **(H)** dominant bacteria in genus level: (a) Clostridia_UCG-014_norank; (b) *Bacteroides*; **(I)** microbiota composition – species level; **(J)** dominant bacteria in species level: (a) *Bacteroides acidifaciens*; (b) *Rikenella microfusus*_DSM_15922; (c) Muribaculaceae_uncultured_barnesiella_sp. The values among groups were compared by Kruskal-Wallis rank sum test, and *P* < 0.05 was considered significant (n = 5). Normal: Normal group; Model: model group; SSP1-H: high dose SSP1 group.

CTX treatment did not significantly alter α-diversity, although the ACE and Chao1 indices showed slight decreases (**Figures 6C, D**). SSP1-H treatment increased these indices by 12.24% and 13.29%, respectively, suggesting enhanced microbial richness.

At the phylum taxonomic rank, CTX administration induced a drop in the relative abundance of Bacteroidetes by 8.76% and increased Firmicutes by 18.4%, resulting in a 75.47% increase in the Firmicutes/Bacteroidetes (F/B) ratio (**Figure 6E**). SSP1-H administration reduced the F/B ratio by 40.68%, consistent with previous reports that Sanghuang polysaccharides can modulate this ratio.

On the genus taxonomic tier, SSP1-H supplementation elevated the proportional abundance of health-promoting bacterial genera including *Bacteroides*, *Alloprevotella*, *Limosilactobacillus*, *Lactobacillus*, and *Alistipes* (**Figure 6F**). The relative abundance of Clostridia_UCG-014_norank was reduced by 62.86% relative to the disease model cohort, whereas the relative abundance of Bacteroides increased from 0.38% in the Model group to 0.55% in the SSP1-H group (**Figure 6G**).

Venn diagram analysis revealed 163 shared OTUs among groups (**Figure 7A**). The SSP1-H group also contained a larger number of unique OTUs, indicating increased microbial diversity. LEfSe analysis further showed that SSP1-H significantly enriched taxa including Bacteroides, Candidatus_Saccharimonas, Odoribacter, and Rikenella (**Figure 7C**).

**Figure 7:**
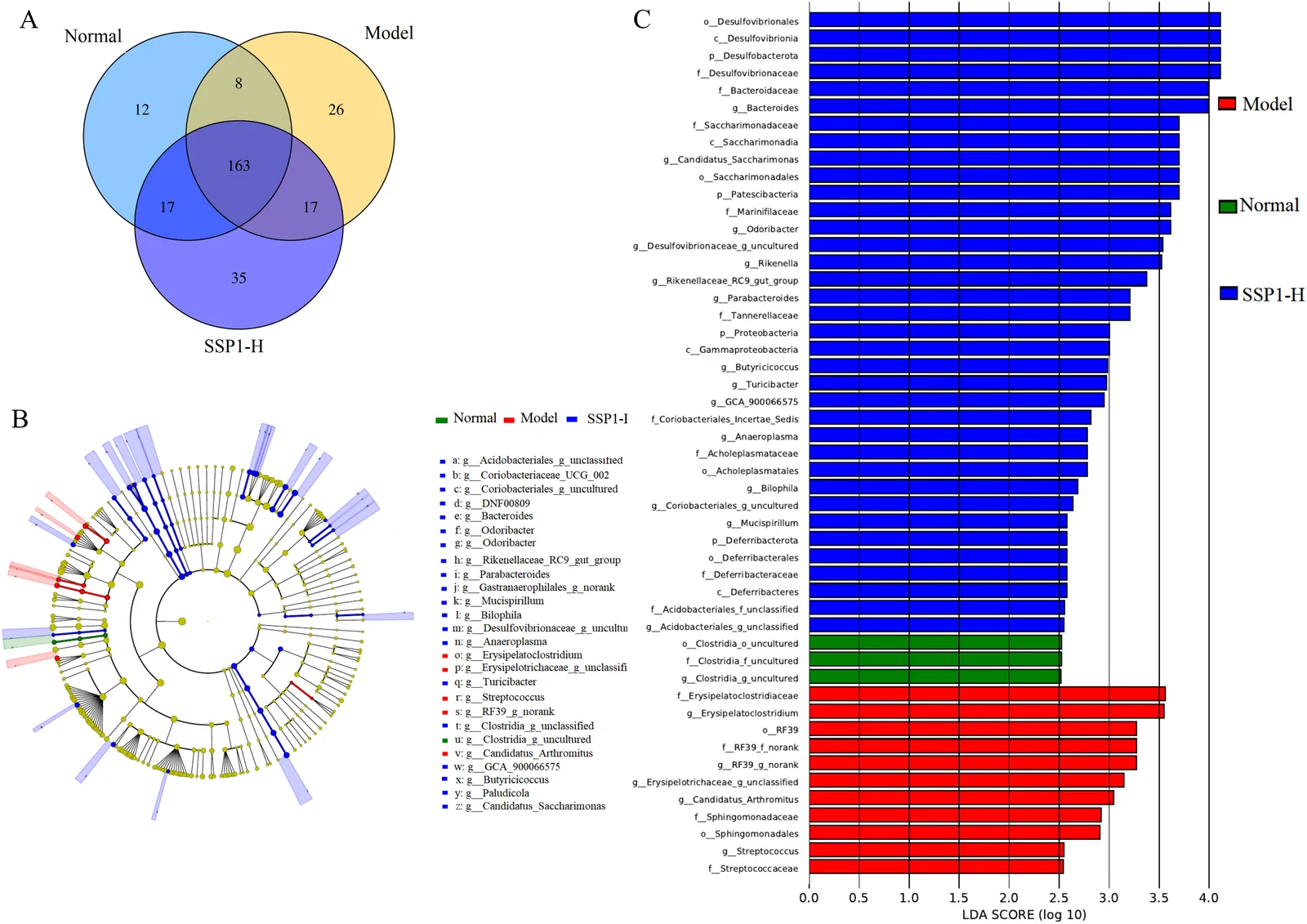
Effects of SSP1-H on gut microbiota structure in fecal. **(A)** Venn diagram; **(B)** The cladogram shows the evolutionary branches of different species; **(C)** The histogram shows the distribution of the LDA values of different species.

### 3.10 SSP1-H increased SCFA production

Gas chromatography was adopted to determine the contents of SCFAs. CTX treatment significantly reduced total SCFA production by 55.19%, with significant decreases in acetic, propionic, butyric, and valeric acids (**Figure 8A**). SSP1-H treatment markedly increased total SCFAs by 70.16% versus the model group. Specifically, propionic acid, acetic acid, and valeric acid increased by 56.09%, 77.40%, and 134.52%, respectively (*P* < 0.05).

**Figure 8:**
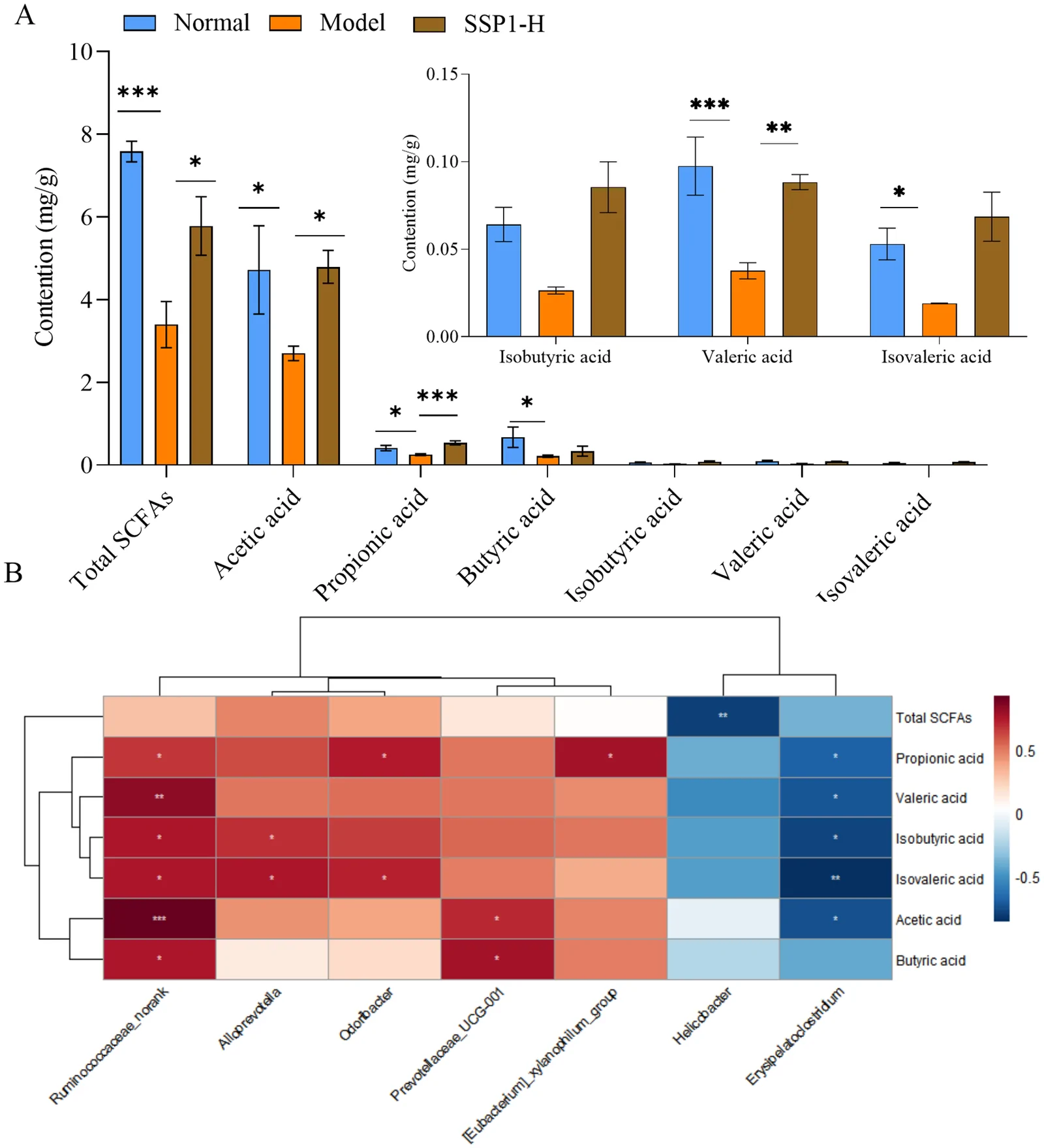
**(A)** concentration of SCFAs in fecal; **(B)** the correlation between gut microbiota and SCFAs. Multiple comparisons were performed by using one-way ANOVA using SPSS 26.0 software. Means with different letters are significantly different (*P* < 0.05) (*n* = 3). Normal: normal group; Model: model group; SSP1-H: high does SSP1 group. *P < 0.05, **P < 0.01, and ***P < 0.001.

Spearman’s rank correlation testing identified prominent linkages spanning bacterial genera and SCFA production (**Figure 8B**). Genera such as Ruminococcaceae_norank, *Alloprevotella*, *Odoribacter*, Prevotellaceae_UCG-001, and [Eubacterium]_xylanophilum_group were positively correlated with SCFAs, whereas Helicobacter and Erysipelatoclostridium showed negative correlations. Further correlation analysis demonstrated that SCFAs were significantly associated with immune and oxidative stress indicators (**Table 2**). SOD, CAT, IL-10, CD19^+^, and CD3^+^ cells were positively correlated with SCFA levels, whereas IL-4, MDA, IL-6, IFN-γ, IL-1β, and TNF-α showed negative correlations.

**Table 2:** The correlation between SCFAs and relative index.

Variable	Total SCFAs	Propionic acid	Valeric acid	Isobutyric acid	Isovaleric acid
MDA	-0.433	-0.667^*^	-0.567	-0.600	-0.483
SOD	0.817^**^	0.600	0.683^*^	0.783^*^	0.967^***^
CAT	0.467	0.833^**^	0.633	0.800^**^	0.717^*^
TNF-α	0.467	0.667^*^	0.533	0.733^*^	0.783^*^
IL-10	0.750^*^	0.700^*^	0.717^*^	0.850^**^	0.933^***^
lymphocytic proliferation	0.467	0.833^**^	0.633	0.800^**^	0.717^*^
CD19^+^	0.917^***^	0.533	0.783^*^	0.750^*^	0.867^**^
CD3^+^	0.917^***^	0.533	0.783^*^	0.750^*^	0.867^**^
IL-2(ConA)	0.400	0.633	0.433	0.667^*^	0.750^*^
IFN-γ(ConA)	-0.883^**^	-0.367	-0.717^*^	-0.550	-0.633
IL-4(ConA)	-0.733^*^	-0.050	-0.500	-0.267	-0.383
IL-1β(LPS)	-0.617	-0.767^*^	-0.683^*^	-0.850^**^	-0.850^**^
IL-6(LPS)	-0.917^***^	-0.533	-0.783^*^	-0.750^*^	-0.867^**^
TNF-α(LPS)	-0.767^*^	-0.367	-0.633	-0.567	-0.717^*^

*P < 0.05, **P < 0.01, ***P < 0.001.

### 3.11 Untargeted metabolomics reveals SSP1-H modulates metabolite profiles

Non-targeted LC-MS/MS metabolome analysis detected 7,314 spectral features in colonic digesta. After metabolite annotation, 256 metabolites were identified as significantly altered after SSP1-H treatment (**Supplementary Table 3**). PLS-DA statistical evaluation yielded evident grouping distinctions among untreated, immunosuppressed and SSP-intervention animal subsets, indicating distinct metabolic profiles (**Figure 9A**). In the Normal vs. Model comparison, 252 metabolites were downregulated and 72 were upregulated (**Supplementary Figure 2A**). In contrast, in the Model vs. SSP1-H comparison, 190 metabolites were upregulated while 50 metabolites were downregulated (**Supplementary Figure 2B**). Meanwhile, core discrepant metabolites were shown in **Figure 9C**.

**Figure 9:**
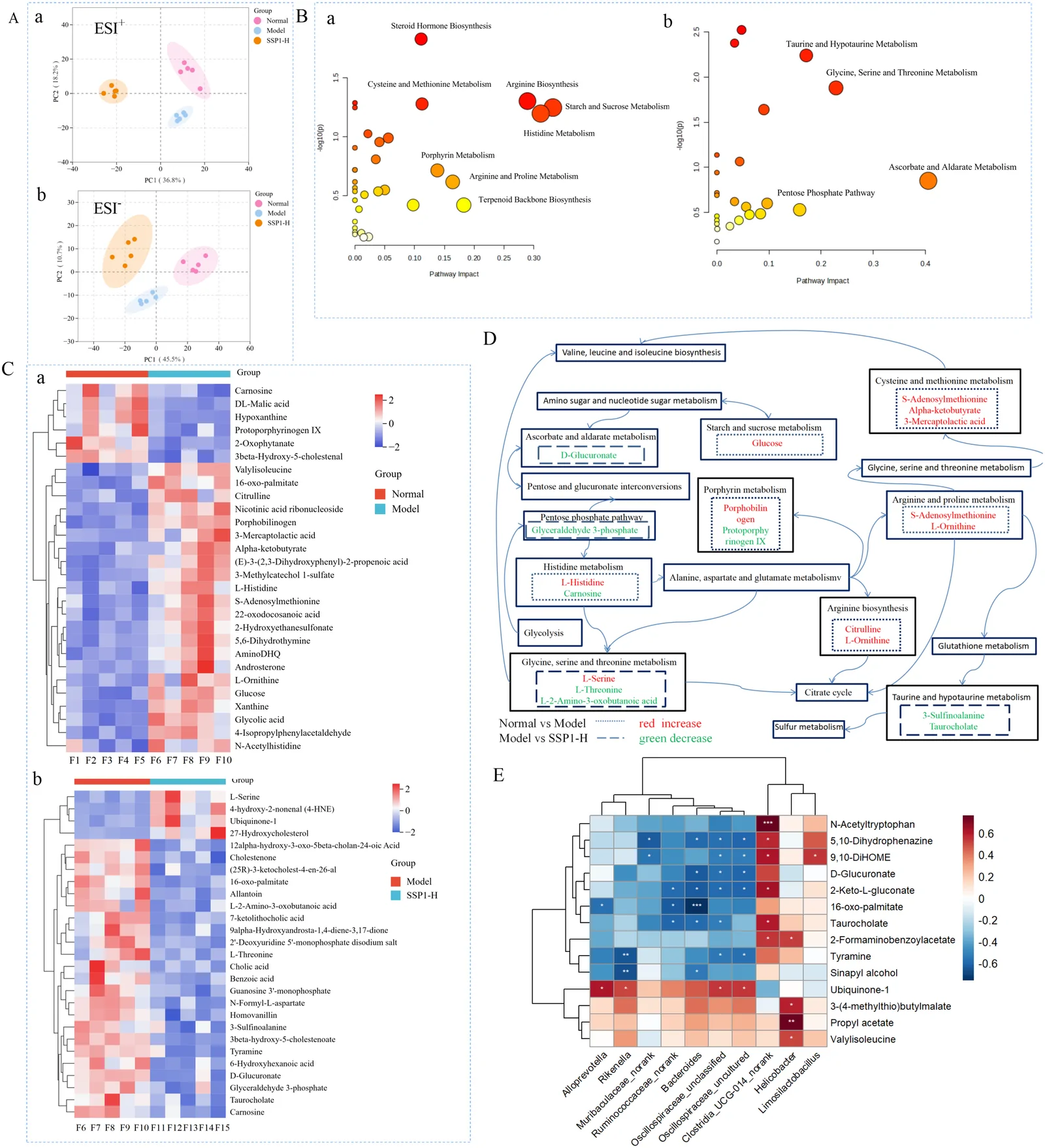
Influence of SSP1-H on the fecal metabolomic signatures of mice. **(A)** Partial least squares discriminant analysis (PLS-DA) scaling plots (N = 5), **(B)** Heatmap of differentially abundant metabolites of the groups (N = 5), **(C)** KEGG pathway enrichment analysis of differentially abundant metabolites in the fecal of different groups (N = 5), **(D)** Relationship between Key Differential Metabolites and Metabolic Pathways in Cyclophosphamide - induced Immunocompromised Mice Treated with SSP1-H, **(E)** the correlation between gut microbiota and metabolites.

KEGG pathway enrichment revealed that CTX-induced metabolic alterations were mainly associated with steroid hormone biosynthesis, arginine biosynthesis, amino acid metabolism, propionate metabolism, and carbohydrate metabolism (**Figure 9B (a)**). After SSP1-H intervention, significantly enriched pathways included the pentose phosphate pathway, ascorbate and aldarate metabolism, and glycine/serine/threonine metabolism (**Figure 9B (b)**). The acquired data imply that SSP1-H confers comprehensive immunomodulatory actions by regulating key metabolic pathways related to amino acid metabolism and carbohydrate metabolism, thereby improving energy supply, maintaining antioxidant balance, and promoting the synthesis of immune-related metabolic intermediates.

Correlation analysis between microbiota and metabolites further revealed strong associations between microbial taxa and metabolic changes. For example, Clostridia_UCG-014_norank, Helicobacter, and Limosilactobacillus exhibited favorable correlation tendencies toward multiple metabolites, exemplified by benzoic acid, taurocholate, and 2-keto-L-gluconate, whereas Ruminococcaceae_norank, Bacteroides, and Oscillospiraceae taxa were negatively correlated with these metabolites (**Figure 9E**).

## 4 Discussion

As an important edible and medicinal resource, Sanghuang has attracted increasing attention because of the immunomodulatory potential of its bioactive components, particularly polysaccharides (17). In this study, SSP1, a polysaccharide isolated from *S. sanghuang*, exhibited a complex monosaccharide composition dominated by galactose, glucose, and mannose. This profile is consistent with previously reported polysaccharides extracted from *Sanghuangporus baumii* (29). Mounting experimental findings demonstrate that polysaccharides derived from fungi possess diverse structural conformations and can regulate host immunity through multi-target interactions (13, 30). To our knowledge, this study systematically demonstrated that oral administration of SSP1-H can effectively alleviate CTX-triggered immunosuppression and gut damage in murine models. Moreover, the protective effects of SSP1-H appear to be linked to the remodeling of the enteric microflora and modulation of microbial metabolites.

CTX is widely used as a chemotherapeutic and immunosuppressive agent. However, it often induces comprehensive systemic immunodeficiency by disrupting lymphocyte development and function, and it is frequently accompanied by intestinal dysbiosis (4, 31). The current project found that SSP1 treatment dose-dependently restored body weight in immunosuppressed mice and significantly increased the thymus and spleen indices, suggesting that SSP1 can effectively alleviate CTX-induced damage to both central and peripheral immune organs. Histopathological observations further confirmed that SSP1, particularly at the high-dose, significantly ameliorated structural damage in the spleen and thymus, reduced inflammatory cell infiltration in the colon, restored goblet cell abundance, thereby maintaining both immune organ architecture and intestinal barrier integrity. The outcomes obtained in the present work are congruent with earlier research reports indicating that plant- and fungal-derived polysaccharides can promote immune organ development and facilitate immune recovery following immunosuppression (1, 32, 33).

Apart from improving immune organ structure, SSP1 significantly enhanced immune cell activity. Specifically, SSP1 promoted LPS-induced splenic lymphocyte proliferation and elevated the percentages of total leukocytes (CD45^+^) and T lymphocytes (CD3^+^) in the spleen. Notably, the immunomodulatory activity of SSP1 exhibited a clear functional polarization. In terms of cytokine production, SSP1 upregulated the synthesis of IL-2 and IL-4 in splenocytes activated by T-cell receptor ligands (anti-CD3/CD28 antibodies) or mitogenic lectins (ConA). Functionally, IL-2 serves as a pivotal immunomodulator that controls T-cell expansion, immune homeostasis, and peripheral immune responses (34), while IL-4 is a canonical Th2-type cytokine (35) involved in humoral immunity. Conversely, SSP1 significantly inhibited the production of IFN-γ, a representative Th1-type cytokine, as well as pro-inflammatory cytokines, namely TNF-α, IL-6 and IL-1β produced by macrophages or B cells following LPS stimulation. Our observations suggest that the immunomodulatory action of SSP1 is not a simple non-specific immune activation. Importantly, SSP1 exerts its regulatory effect mainly via suppressing excessive Th1-mediated inflammatory responses, rather than driving further Th2 polarization by increasing IL-4. Instead of straightforward Th2-type immune skewing, SSP1 remodels the disrupted Th1/Th2 balance in CTX-immunosuppressed mice. Such a regulatory pattern may help restore immune balance while preventing tissue damage caused by excessive immune activation, which may provide potential benefits for immune reconstitution following chemotherapy. We acknowledge the complexity of this Th1/Th2 remodeling: Th1 responses are indispensable for anti-tumor immunity and defense against intracellular pathogens, yet uncontrolled Th1 hyperactivation can trigger inflammatory injury during immune recovery (36). Similar regulatory effects on the Th1/Th2 balance have also been reported for other bioactive polysaccharides (37). In addition, the present study did not detect Treg-associated cytokines such as TGF-β; further investigation incorporating these markers will help clarify whether regulatory T cells participate in SSP1-mediated immunomodulation.

Over the past decade, the crosstalk between gut commensal microbes and the immune system has been recognized as a key modulator of the systemic effects of natural products (12, 38, 39). As indigestible dietary fiber, polysaccharides serve as important substrates for intestinal microbes (40), and their supplementation can substantially reshape microbial community structure (41). In this study, 16S rRNA sequencing showed that although the α-diversity of the gut microbiota remained largely unchanged following CTX exposure, the treatment significantly altered the microbial profile, notably reducing the Bacteroidetes/Firmicutes ratio. The F/B ratio is widely regarded as an indicator of microbial homeostasis, and its elevation usually reflects microbiota dysbiosis. Increased F/B ratio have also been reported in several inflammatory diseases, including psoriasis (42). Mirroring these results, the CTX intervention markedly increased the F/B ratio in mice, while SSP1-H administration effectively reversed this trend. More importantly, SSP1-H selectively enriched several potential beneficial microbial taxa, including *Bacteroides*, *Bacteroides acidifaciens* and *Rikenella*. This microbial regulatory pattern is consistent with previous reports describing the modulatory impact of Sanghuang polysaccharides on the intestinal flora of CTX-treated immunosuppressed mice (43). LEfSe analysis further identified *Bacteroides* and *Odoribacter* as signature taxa in the SSP1-H group, suggesting that such microbial taxa could play a central part in mediating the physiological effects of SSP1-H.

Interestingly, several enriched microbial taxa, including *Odoribacter* and *Alloprevotella*, exhibited strong positive associations with the restoration of SCFA levels in cecal contents. SCFAs are key immunomodulatory metabolites produced by microbial fermentation of dietary fibers (44). Of these, propionic acid, acetic acid, and butyric acid are the primary SCFAs involved in immune regulation (45). Acetate has been shown to modulate macrophage polarization and enhance the cytotoxic function of CD8^+^ T cells (46). Propionate inhibits pro-inflammatory T helper cell responses (e.g., Th1 and Th17) while enhancing regulatory T-cell function, thereby maintaining immune homeostasis (44). Such immune balance is particularly important for controlling the hyperactive immune responses observed in autoimmune diseases such as multiple sclerosis (MS) (47). In this study, SSP1-H treatment significantly boosted the production of total SCFAs, valeric acid, propionic acid and acetic acid. Moreover, SCFA levels were positively correlated with antioxidant indicators, T lymphocytes (CD3^+^) and the anti-inflammatory cytokine IL-10, while exhibiting strong inverse associations with pro-inflammatory mediators, including TNF-α. These findings suggest that SSP1-H may exert prebiotic-like effects, characterized by the enrichment of potentially beneficial microbial taxa and increased SCFA production, which may contribute to the systemic immunomodulatory effects observed in this study.

To further elucidate the metabolic mechanism underlying SSP1-H intervention, untargeted metabolomics was performed. KEGG enrichment analysis further revealed that SSP1-H significantly affected multiple metabolic pathways, including glycine, serine and threonine metabolism, taurine and hypotaurine metabolism, ascorbic acid and aldarate metabolism; and the pentose phosphate pathway. Within this metabolic network, N-formyl-L-aspartic acid may indirectly regulate glycine, serine and threonine metabolism by its involvement in alanine, aspartate and glutamate metabolism. L-serine is known to be an essential metabolite supporting B-cell proliferation and antibody production, and its deficiency can suppress humoral immunity (48). Meanwhile, fluctuations in L-2-amino-3-oxobutanoic acid, a metabolic intermediate of L-threonine, may influence memory T-cell differentiation through the mTORC1 signaling pathway (49). In this study, SSP1-H coordinately upregulated L-serine levels while downregulating L-threonine and its metabolic intermediates, revealing potential correlative metabolic signatures that may contribute to its dual effect of enhancing humoral immunity while regulating cellular immune response. Furthermore, taurine and hypotaurine metabolism was also significantly altered. Taurocholic acid, the end product of this pathway, has been proven to inhibit macrophage phagocytosis and attenuate delayed-type hypersensitivity response (50). The reduction of taurocholate levels following SSP1-H treatment may therefore help ameliorate immunosuppression by relieving its inhibitory effects on both innate and adaptive immunity.

In addition, both the pentose phosphate pathway and ascorbic acid metabolism were activated following SSP1-H intervention. The pentose phosphate pathway provides nucleotide precursors and NADPH necessary for immune cell proliferation, whereas ascorbic acid metabolism contributes to maintaining redox balance. Together, these metabolic changes may support immune cell activation and function recovery. Notably, redox homeostasis serves as a critical bridge connecting oxidative stress, cellular senescence and immune competence. Chemotherapy-induced oxidative damage can trigger therapy-induced senescence and the release of SASP factors, further exacerbating immunosenescence and immune dysfunction (51). In the present model, SSP1-H increased antioxidant enzyme activities and reduced MDA accumulation, implying that its antioxidant property may interrupt the vicious cycle between oxidative injury and impaired immune function. Strategies targeting oxidative stress to alleviate chemotherapy-related immunosenescence offer promising directions for developing adjuvant agents, which highlights the translational prospect of SSP1-H.

Importantly, statistical correlation analysis revealed significant links between certain gut bacteria and key metabolites, suggesting a potential correlative “microbiota–metabolism–immunity” regulatory network. For example, microbial genera such as *Clostridia_UCG−014* were positively correlated with taurocholic acid, a metabolite with immunosuppressive properties, whereas beneficial bacteria including *Bacteroides* exhibited a negative correlation, consistent with previous reports (52). These results indicate that SSP1-H treatment is correlated with reduced taurocholic acid levels alongside remodeled gut microbiota composition, suppressing bacteria associated with immunosuppressive metabolites while promoting beneficial taxa. These findings are consistent with the possibility that SSP1-H-induced microbiota remodeling and metabolic changes contribute to an intestinal microenvironment favorable for immune recovery.

However, this study still has certain limitations. First, Importantly, our data reveal correlative associations between gut microbial shifts, metabolic alterations, and immune improvement rather than definitive causal relationships. Although the enrichment of SCFA-producing taxa, increased SCFA concentrations, and their associations with immune and oxidative stress parameters support a potential microbiota–metabolism–immune axis, these findings do not establish that microbial remodeling or SCFAs are necessary mediators of SSP1-induced immune recovery. Further mechanistic studies, including fecal microbiota transplantation, selective SCFA supplementation or depletion, and microbiota-depletion approaches, are required to determine whether SSP1-induced microbial remodeling and SCFA production are causally responsible for the observed immune protection. Second, whether SSP1 contains specific active polysaccharide fragments responsible for the observed effects remains unclear and requires further purification and identification. Third, the impacts of SSP1 on intestinal local immune cells, such as lamina propria lymphocytes, remain to be investigated. Fourth, although the monosaccharide composition of SSP1 has been reported previously, this study lacks comprehensive structural characterization, including molecular weight distribution, glycosidic linkage patterns, and backbone structural information obtained by methylation analysis or NMR spectroscopy. Since the biological activity of polysaccharides is strongly dependent on these structural parameters, insufficient structural data restricts the exploration of the structure-activity relationship of SSP1 and hinders direct comparative analysis with other immunomodulatory polysaccharides. Future studies should therefore focus on characterizing the fine chemical structure of SSP1, identifying its active components and evaluating its therapeutic potential in additional disease models.

## 5 Conclusion

In summary, this research demonstrates that SSP1, a polysaccharide harvested from *S. sanghuang*, effectively ameliorates CTX-induced immunosuppression in mice. The immunomodulatory function of SSP1-H occurs at both the intestinal mucosa and the systemic immune level, involving coordinated remodeling of the microbiota-metabolism-immunity axis. Mechanistically, SSP1-H regulates immune cell function. The immune recovery after SSP1-H intervention is accompanied by remodeled gut microbiota, increased SCFA concentration, reduced levels of metabolites potentially involved in immune regulation, including taurocholic acid, and altered host-microbe metabolic pathways such as serine, threonine and taurine metabolism. Present experimental outcomes point to SSP1-H’s prospective use as adjunct agent alongside chemo treatment in relieving immune impairment and a functional dietary supplement for preserving immune steady-state. Collectively, these findings provide experimental evidence supporting the potential of SSP1 as a functional food ingredient or adjunctive nutritional strategy for maintaining immune homeostasis during chemotherapy-associated immunosuppression.

## Data Availability

The datasets presented in this study can be found in online repositories. The names of the repository/repositories and accession number(s) can be found in the article/**Supplementary Material**.
