# Supplementary material for: Immunomodulatory effects of Sanghuangporus sanghuang polysaccharide SSP1 in cyclophosphamide-induced immunosuppressed mice: associations with gut microbiota, short-chain fatty acids, and metabolic remodeling
Source: Front Immunol. 2026 Sep 17;17:1931873. doi: 10.3389/fimmu.2026.1931873 (PMC13627069; doi:10.3389/fimmu.2026.1931873)
Supplement: Supplementary file 1 [file Supplementaryfile1.docx]

The raw 16S rRNA sequencing data have been deposited in the NCBI BioProject database under accession number PRJNA1394512 (available at:<https://www.ncbi.nlm.nih.gov/bioproject/PRJNA1394512>）

**Table S1** Effects of different doses of SSP1 on the body weight of mice

| Group | DOSE（mg/kg·bw） | | | 1 day | | 4 day | | 10 day | | 16 day | |
| --- | --- | --- | --- | --- | --- | --- | --- | --- | --- | --- | --- |
|  |  |  |  | n | Weight/g | n | Weight/g | n | Weight/g | n | Weight/g |
| Normal | | — | 10 | | 18.57±0.96 | 10 | 19.30±1.02 | 10 | 20.91±0.61 | 10 | 22.39±0.65 |
| Model | | — | 10 | | 18.94±1.63 | 10 | 15.80±1.13^***^ | 10 | 18.44±0.89^**^ | 10 | 19.08±0.88^***^ |
| Control | | 40 | 10 | | 18.87±1.42 | 10 | 16.92±1.19^**^ | 10 | 19.33±1.48^*^ | 10 | 20.72±1.44^**##^ |
| SSP1-L | | 100 | 10 | | 19.08±1.27 | 10 | 17.59±1.31^*##^ | 10 | 17.40±1.51^***^ | 10 | 19.79±0.92^***^ |
| SSP1-M | | 200 | 10 | | 18.96±1.27 | 10 | 17.30±1.51^*#^ | 10 | 18.68±0.90^**^ | 10 | 20.10±1.08^***^ |
| SSP1-H | | 400 | 10 | | 18.71±0.96 | 10 | 18.03±1.76^###^ | 10 | 19.05±1.12^**^ | 10 | 21.14±0.78^##^ |

Note： **P* < 0.05, **P* < 0.01 and ***P* < 0.001 vs. the Normal group; ^#^*P* < 0.05, ^##^*P* < 0.01 and ^###^*P* < 0.001 vs. the Model group.

**Fig. S1** Representative graph of flow cytometry assay: from left to right, normal, model, control, SSP1-L, SSP1-M and SSP1-H groups; A: F4/80; B: CD45; C: CD3^-^/CD19^-^, CD3^+^, CD19^+^

**Table S2** Sequence number of samples

| Samples | Sequence number | Samples | Sequence number |
| --- | --- | --- | --- |
| F1 | 93225 | F9 | 92710 |
| F2 | 94726 | F10 | 92405 |
| F3 | 96612 | F11 | 53300 |
| F4 | 96475 | F12 | 91750 |
| F5 | 92735 | F13 | 93886 |
| F6 | 94247 | F14 | 94807 |
| F7 | 93257 | F15 | 95269 |
| F8 | 78947 |  |  |

Note：F1-F5（Normal Group）；F6-F10（Model Group）；F26-F30（SSP1-H Group）

**Fig. S2** The volcano plot shows the differential metabolites between treatment groups (red dot: significantly up-regulated metabolites, blue dot: significantly down-regulated metabolites, gray dot: the metabolites with no significant difference, Q < 0.05, VIP>1).

| **Table S3** Significantly changed metabolites in Normal, Model and SSP1-H group | | | | | | | |
| --- | --- | --- | --- | --- | --- | --- | --- |
| **ID** | **name** | **mz** | **formula** | **P.value** | **-log10**  **(P.value)** | **FDR** | **VIP** |
| M112T70_1 | 2-Ethyl-4-methyloxazole | 112.0397 | C6H9NO | 0.021207895 | 1.67 | 0.272988177 | 1.000110287 |
| M113T52_2 | (S)-2-Piperazinecarboxylate | 113.0714 | C5H10N2O2 | 0.017332932 | 1.76 | 0.251999467 | 2.14006259 |
| M118T54 | L-2-Amino-3-oxobutanoic acid | 118.0863 | C4H7NO3 | 0.000243381 | 3.61 | 0.025674183 | 2.790867884 |
| M134T252 | Indolin-2-one | 134.0599 | C8H7NO | 0.045713887 | 1.34 | 0.365412283 | 1.043980463 |
| M144T122 | Ethyl hydrogen sulfate | 144.1385 | C2H6O4S | 0.005028078 | 2.3 | 0.144765088 | 2.157072436 |
| M144T141_2 | Tryptamine | 144.081 | C10H12N2 | 0.001078859 | 2.97 | 0.061497792 | 2.028742508 |
| M149T256 | Phthalic anhydride | 149.0597 | C8H4O3 | 0.031578915 | 1.5 | 0.311235313 | 1.70040546 |
| M152T55_1 | Norepinephrine | 152.0701 | C8H11NO3 | 0.041142435 | 1.39 | 0.35109093 | 1.945938487 |
| M153T44 | Glycolic acid | 153.0328 | C2H4O3 | 0.003665552 | 2.44 | 0.125217209 | 1.521155225 |
| M154T54_2 | 2,4-Diamino-6-hydroxylaminotoluene | 154.0966 | C7H11N3O | 0.035465292 | 1.45 | 0.327885818 | 2.095631289 |
| M160T67 | cis-3-Hexenyl acetate | 160.0608 | C8H14O2 | 0.001777118 | 2.75 | 0.083308112 | 2.656755229 |
| M162T334 | (R)-Boschniakine | 162.054 | C10H11NO | 0.034431564 | 1.46 | 0.321899436 | 1.160591972 |
| M165T443 | 1,5-Anhydro-d-mannitol | 165.1273 | C6H12O5 | 0.010692818 | 1.97 | 0.215417568 | 1.506626154 |
| M168T55_1 | 4-Coumaryl alcohol | 168.1021 | C9H10O2 | 0.012979093 | 1.89 | 0.228256045 | 1.821491599 |
| M172T70 | 1-Isothiocyanato-6-(methylthio)hexane | 172.1083 | C8H15NS2 | 0.029927207 | 1.52 | 0.302020514 | 1.878356321 |
| M175T336 | 2,4,6-Triethyl-1,3,5-trioxane | 175.1122 | C9H18O3 | 0.030491225 | 1.52 | 0.303376257 | 1.358768501 |
| M176T52 | Citrulline | 176.1031 | C6H13N3O3 | 0.011181873 | 1.95 | 0.219113566 | 1.732340718 |
| M176T53 | Argininic acid | 176.0921 | C6H13N3O3 | 0.009678664 | 2.01 | 0.206355886 | 2.580318511 |
| M180T339 | 2-Amino-3-phenylbutanoic acid | 180.1022 | C10H13NO2 | 0.036958522 | 1.43 | 0.332036454 | 1.792370061 |
| M182T200 | 4-Hydroxycinnamic acid | 182.08 | C9H8O3 | 0.017805304 | 1.75 | 0.253677565 | 1.021926389 |
| M182T55_2 | Thymol methyl ether | 182.1179 | C11H16O | 0.032420335 | 1.49 | 0.315279135 | 1.49305407 |
| M187T70_2 | ACMC-20lajd | 187.1082 | C8H16N2O4 | 3.51044E-06 | 5.45 | 0.001100696 | 2.2753068 |
| M189T51 | 7,8-diaminopelargonate | 189.1602 | C9H20N2O2 | 0.023717937 | 1.62 | 0.27966122 | 1.475057369 |
| M190T162 | 2-Hydroxy-4-quinolincarboxylic acid | 190.0505 | C10H7NO3 | 0.003798165 | 2.42 | 0.126754996 | 2.420024196 |
| M192T54 | Valienone | 192.0879 | C7H10O5 | 0.008989909 | 2.05 | 0.198619952 | 1.834444273 |
| M193T425 | (6E)-8-hydroxygeraniol | 193.1225 | C10H18O2 | 0.013109194 | 1.88 | 0.228256045 | 1.488785836 |
| M194T113 | EDDA | 194.1161 | C6H12N2O4 | 0.028428625 | 1.55 | 0.297508216 | 2.091110051 |
| M200T196 | N-Formyl-L-aspartate | 200.1281 | C5H7NO5 | 0.011095745 | 1.95 | 0.218126832 | 1.662118591 |
| M200T55_2 | gamma-Glutamyl-beta-aminopropiononitrile | 200.1034 | C8H13N3O3 | 0.013620498 | 1.87 | 0.231853556 | 1.38038562 |
| M201T54_1 | Serylisoleucine | 201.1234 | C9H18N2O4 | 0.00316668 | 2.5 | 0.120146987 | 2.301084344 |
| M210T54_2 | Carnosine | 210.1126 | C9H14N4O3 | 0.00199519 | 2.7 | 0.089514241 | 1.318136829 |
| M211T264 | Aprobarbital | 211.1336 | C10H14N2O3 | 0.006755638 | 2.17 | 0.173671284 | 2.299888528 |
| M214T59 | 2-amino-3-oxo-4-(phosphonooxy)butanoic acid | 213.983 | C4H8NO7P | 0.006538342 | 2.18 | 0.171996026 | 2.375558725 |
| M219T51 | 5,6-Dihydro-4-methoxy-6-[2-(4-methoxyphenyl)ethyl]-2H-pyran-2-one | 219.0351 | C15H18O4 | 3.61229E-06 | 5.44 | 0.001100696 | 1.255353084 |
| M227T295 | Rhodinyl butyrate | 227.128 | C14H26O2 | 7.65301E-07 | 6.12 | 0.000310925 | 2.228844407 |
| M228T70_2 | Sinapyl alcohol | 228.1233 | C11H14O4 | 7.91356E-08 | 7.1 | 5.64607E-05 | 3.311777308 |
| M232T326 | Aminophenazone | 232.1336 | C13H17N3O | 0.000167774 | 3.78 | 0.020851571 | 2.468168033 |
| M232T54_1 | gamma-Aminobutyryllysine | 232.0819 | C10H21N3O3 | 0.012873287 | 1.89 | 0.228256045 | 1.255770208 |
| M233T70_2 | Costunolide | 233.1495 | C15H20O2 | 0.011279926 | 1.95 | 0.219300082 | 1.623603997 |
| M251T209 | 3beta-Dihydroxymarasmene | 251.1754 | C15H22O3 | 4.85822E-05 | 4.31 | 0.009349507 | 2.740737189 |
| M258T105 | 4a-Hydroxytetrahydrobiopterin | 258.17 | C9H15N5O4 | 0.020042487 | 1.7 | 0.265248209 | 1.962108007 |
| M260T157 | 4-Hydroxycinnamoylagmatine | 260.1034 | C14H20N4O2 | 0.031074676 | 1.51 | 0.307925613 | 1.355411058 |
| M261T150 | Biotin sulfoxide | 261.1448 | C10H16N2O4S | 0.004757861 | 2.32 | 0.14377783 | 1.087079016 |
| M265T244_1 | Istanbulin A | 265.1435 | C15H20O4 | 1.86222E-07 | 6.73 | 0.000104757 | 1.996156638 |
| M267T110 | 4-O-dimethylallyl-L-tyrosine | 267.1704 | C14H19NO3 | 0.005757922 | 2.24 | 0.157706678 | 1.810292384 |
| M272T85 | 2,4-Dodecadienoic acid pyrrolidide | 272.1604 | C16H27NO | 0.021214993 | 1.67 | 0.272988177 | 1.216676871 |
| M279T258 | Leucylphenylalanine | 279.1706 | C15H22N2O3 | 0.03267265 | 1.49 | 0.315634204 | 1.026505139 |
| M283T229 | Oryzalexin C | 283.2018 | C20H28O2 | 0.01609786 | 1.79 | 0.2468001 | 1.799553951 |
| M288T52 | Rutaecarpine | 288.1072 | C18H13N3O | 0.015414603 | 1.81 | 0.242923427 | 1.577280212 |
| M288T55_1 | Arginylhydroxyproline | 288.1553 | C11H21N5O4 | 0.035201916 | 1.45 | 0.32668986 | 1.378812334 |
| M293T434 | Docosadienoate (22_2n6) | 293.2478 | C22H40O2 | 0.001502892 | 2.82 | 0.075484178 | 1.425051111 |
| M295T306_2 | 12,13-Epoxy-9,15-octadecadienoic acid | 295.1525 | C18H30O3 | 0.006697632 | 2.17 | 0.173671284 | 2.738041452 |
| M298T230 | pisatin | 298.1315 | C17H14O6 | 0.0013831 | 2.86 | 0.072247214 | 1.980731463 |
| M300T196 | TETRAHYDROPAPAVERINE | 300.1913 | C20H25NO4 | 0.009186819 | 2.04 | 0.200064174 | 2.48263 |
| M300T228 | protoemetine | 300.1975 | C19H27NO3 | 0.017710263 | 1.75 | 0.253677565 | 1.334110524 |
| M300T55 | Aminoparathion | 300.1554 | C10H16NO3PS | 0.016458584 | 1.78 | 0.247691972 | 1.160889885 |
| M302T120 | Boldione | 302.2089 | C19H24O2 | 0.005447696 | 2.26 | 0.15263986 | 1.822848005 |
| M302T49 | Prolyl-Tryptophan | 302.2435 | C16H19N3O3 | 0.014086088 | 1.85 | 0.23371535 | 2.45891657 |
| M306T214 | Dihydrochelerythrine | 306.1453 | C21H19NO4 | 0.003747251 | 2.43 | 0.125704803 | 1.955187222 |
| M310T361 | ST 19_0;O2 | 310.2746 | C19H32O2 | 0.032186966 | 1.49 | 0.313844376 | 2.017273998 |
| M312T271 | MG(0_0_16_1(9Z)_0_0) | 312.2262 | C19H36O4 | 0.041721356 | 1.38 | 0.353543772 | 1.053199186 |
| M313T306 | ST 21_4;O2 | 313.2162 | C21H28O2 | 0.034860042 | 1.46 | 0.32475848 | 1.567519956 |
| M314T117 | Crocetindial | 314.2071 | C20H24O2 | 0.022178027 | 1.65 | 0.273043618 | 1.466313411 |
| M318T252 | 19-Oxoandrost-4-ene-3,17-dione | 318.1927 | C19H24O3 | 0.014734277 | 1.83 | 0.236817065 | 1.065247877 |
| M319T315 | 15-HPETE | 319.2274 | C20H32O4 | 0.03528249 | 1.45 | 0.327022624 | 2.436437973 |
| M321T260 | Scopolamine | 321.1804 | C17H21NO4 | 0.024653705 | 1.61 | 0.284822341 | 1.497719255 |
| M322T72 | Famciclovir | 322.1403 | C14H19N5O4 | 0.027753239 | 1.56 | 0.297508216 | 2.293164093 |
| M325T110 | 5-Amino-2,3-dihydro-6-(3-hydroxy-4-methoxy-1-oxobutyl)-2,2-dimethyl-4H-1-benzopyran-4-one | 325.1505 | C16H21NO5 | 0.000812363 | 3.09 | 0.052573516 | 2.069442724 |
| M326T111 | Hexyl 2,5-dichlorophenylphosphoroamidate | 326.0378 | C12H18Cl2NO3P | 0.011714028 | 1.93 | 0.223667581 | 2.038843719 |
| M327T363 | FA 20_3;O4 | 327.2537 | C20H34O6 | 0.026646082 | 1.57 | 0.296413213 | 1.321355784 |
| M330T303 | 2,2,6,10,14-Pentamethylpentadecanoic acid | 330.2641 | C20H40O2 | 0.028364998 | 1.55 | 0.297508216 | 1.068033974 |
| M331T54_2 | Ephedrine | 331.2339 | C10H15NO | 0.026725665 | 1.57 | 0.296577828 | 1.077720229 |
| M332T322 | pregn-5-ene-3,20-dione | 332.2583 | C21H30O2 | 0.033568431 | 1.47 | 0.318387549 | 2.336374914 |
| M341T222 | Perindoprilat | 341.2075 | C17H28N2O5 | 0.008210416 | 2.09 | 0.187049145 | 2.388134595 |
| M344T405 | 2-Oxophytanate | 344.3166 | C20H38O3 | 0.004320934 | 2.36 | 0.137386907 | 1.027007343 |
| M349T205 | 12,20-Dioxo-leukotriene B4 | 349.1867 | C20H28O5 | 0.000538178 | 3.27 | 0.040822793 | 2.980357932 |
| M349T275 | Sinapine | 349.2591 | [C16H24NO5]+ | 0.030401024 | 1.52 | 0.303376257 | 2.320615815 |
| M351T226 | Serotinose | 351.203 | C11H20O10 | 0.042805794 | 1.37 | 0.356942728 | 1.222108234 |
| M352T273_1 | Meloxicam | 352.176 | C14H13N3O4S2 | 0.01787044 | 1.75 | 0.253677565 | 1.20608912 |
| M353T395 | Portuloside A | 353.2687 | C16H26O7 | 0.014213187 | 1.85 | 0.234101438 | 2.103228887 |
| M353T420 | episterone | 353.3527 | C28H44O | 0.021359612 | 1.67 | 0.272988177 | 2.32173827 |
| M355T268 | Artocarpesin | 355.1731 | C20H18O6 | 0.032623938 | 1.49 | 0.315580504 | 1.733389971 |
| M363T224 | 2-Amino-5-[[1-[carboxymethyl(2-hydroxypropanoyl)amino]-1-oxo-3-sulfanylpropan-2-yl]amino]-5-oxopentanoic acid | 363.2576 | C13H21N3O8S | 2.8756E-05 | 4.54 | 0.006590401 | 2.813792743 |
| M364T268 | Guanosine 3'-monophosphate | 364.2551 | C10H14N5O8P | 0.013914817 | 1.86 | 0.23371535 | 1.891476072 |
| M366T425 | 5-Heptadecyl-1,3-benzenediol | 366.3004 | C23H40O2 | 0.045525436 | 1.34 | 0.365052101 | 1.589844301 |
| M367T271 | Atovaquone | 367.2278 | C22H19ClO3 | 0.033371611 | 1.48 | 0.317978042 | 1.655211525 |
| M367T363 | Cholestenone | 367.3322 | C27H44O | 0.007740232 | 2.11 | 0.182594574 | 2.235612499 |
| M373T308_2 | 12alpha-hydroxy-3-oxo-5beta-cholan-24-oic Acid | 373.2732 | C24H38O4 | 0.016481607 | 1.78 | 0.247691972 | 1.683125941 |
| M376T252 | Oxidized Cypridina luciferin | 376.2229 | C21H27N7O | 0.002350271 | 2.63 | 0.098938905 | 2.299294139 |
| M386T278 | Terminaline | 386.3018 | C23H41NO2 | 0.019383758 | 1.71 | 0.261056029 | 1.241113326 |
| M387T339_2 | 8-8'-Dehydrodiferulic acid | 387.3575 | C20H18O8 | 0.001238214 | 2.91 | 0.068598914 | 2.082514338 |
| M388T220 | ST 24_3;O5 | 388.2542 | C24H36O5 | 0.044775211 | 1.35 | 0.362614746 | 1.103573083 |
| M392T356 | 3alpha,7alpha,12beta-Trihydroxy-5beta-cholan-24-oic Acid | 392.2882 | C24H40O5 | 0.000630929 | 3.2 | 0.043528147 | 1.880670698 |
| M399T389 | (25R)-3-ketocholest-4-en-26-al | 399.3281 | C27H42O2 | 0.002565601 | 2.59 | 0.105405844 | 2.366719895 |
| M400T431_1 | 3beta-hydroxy-5-cholestenoate | 400.3319 | C27H44O3 | 0.000167677 | 3.78 | 0.020851571 | 2.678216066 |
| M405T287 | (E)-4'-Methylresveratrol 3-glucoside | 405.2643 | C21H24O8 | 0.028079963 | 1.55 | 0.297508216 | 2.29159581 |
| M408T378 | 7-ketolithocholic acid | 408.3103 | C24H38O4 | 0.037605622 | 1.42 | 0.334969445 | 1.709211914 |
| M411T257 | Sesamolinol | 411.2488 | C20H20O7 | 8.74222E-05 | 4.06 | 0.014324868 | 3.040659759 |
| M411T412 | avenastenone | 411.3593 | C29H46O | 0.02720848 | 1.57 | 0.297508216 | 1.34039233 |
| M413T443 | ST 29_2;O | 413.3785 | C29H48O | 0.010979002 | 1.96 | 0.217815905 | 2.569877445 |
| M420T201 | Roquefortine F | 420.2048 | C23H25N5O3 | 0.008208649 | 2.09 | 0.187049145 | 2.366215194 |
| M421T357 | ST 28_2;O | 421.3465 | C28H46O | 0.024565123 | 1.61 | 0.284248018 | 2.011570137 |
| M423T393 | 3beta-Hydroxy-5-cholestenal | 423.3262 | C27H44O2 | 0.02150481 | 1.67 | 0.272988177 | 1.61809391 |
| M429T314 | Spirotaccagenin | 429.1871 | C27H42O5 | 0.033079232 | 1.48 | 0.317204695 | 1.858740987 |
| M432T338 | SCHEMBL16431292 | 432.3328 | C30H38O | 0.023824551 | 1.62 | 0.27966122 | 1.373969469 |
| M437T364 | Demethylphylloquinone | 437.3414 | C30H44O2 | 0.01133756 | 1.95 | 0.219300082 | 1.84965751 |
| M437T51 | Tianeptine | 437.1251 | C21H25ClN2O4S | 0.019320589 | 1.71 | 0.261056029 | 2.297918458 |
| M438T238 | 20-Hydroxy-leukotriene E4 | 438.2236 | C23H37NO6S | 0.00132963 | 2.88 | 0.070460746 | 2.23197872 |
| M439T306_2 | Campesterol | 439.3195 | C28H48O | 0.013012922 | 1.89 | 0.228256045 | 1.587071658 |
|  |  |  |  |  |  |  |  |
| M439T357 | (+-)-Ethyl 3-hydroxy-2-methylbutyrate | 439.3565 | C30H48O3 | 0.000571903 | 3.24 | 0.041823266 | 2.413310713 |
| M441T442 | 3beta-Hydroxycucurbita-7,24-dien-11-one | 441.3721 | C30H48O2 | 0.007511102 | 2.12 | 0.180810126 | 2.478321206 |
| M442T233 | glandicoline A | 442.256 | C22H21N5O3 | 0.023931625 | 1.62 | 0.279867675 | 1.051795257 |
| M442T234 | Roquefortine L | 442.2703 | C22H21N5O3 | 0.000758526 | 3.12 | 0.050428155 | 2.80319389 |
| M442T275 | N,N-Diacetylchitobiose | 442.2056 | C16H28N2O11 | 0.017853963 | 1.75 | 0.253677565 | 2.057630155 |
| M444T432 | Protopanaxadiol | 444.3687 | C30H52O3 | 0.000564217 | 3.25 | 0.041677969 | 1.725479131 |
| M445T421 | Schleichol 1 | 445.329 | C30H52O2 | 0.000534452 | 3.27 | 0.040822793 | 1.256803409 |
| M447T445 | ST 24_1;O5 | 447.3819 | C24H40O5 | 0.018407876 | 1.73 | 0.257770597 | 1.993804633 |
| M450T443 | ST 28_1;O3 | 450.3919 | C28H48O3 | 0.040921489 | 1.39 | 0.350420195 | 1.11598996 |
| M471T366 | Didodecyl thiobispropanoate | 471.3074 | C30H58O4S | 0.018829355 | 1.73 | 0.260300701 | 1.784500108 |
| M494T381 | LysoPC(16_1(9Z)_0_0) | 494.3242 | C24H48NO7P | 0.000588984 | 3.23 | 0.042227838 | 2.731928032 |
| M505T322 | PA(10_0_13_0) | 505.3668 | C26H51O8P | 0.032616487 | 1.49 | 0.315580504 | 1.714132176 |
| M600T446 | Oryzanol C | 600.4176 | C41H60O4 | 0.000541476 | 3.27 | 0.040822793 | 2.184653225 |
| M607T377 | Rhodovibrin | 607.4547 | C41H60O2 | 0.025141993 | 1.6 | 0.288187138 | 1.18292419 |
| M627T443 | PA(14_0_18_3(9Z,12Z,15Z)) | 626.5025 | C35H63O8P | 0.013409416 | 1.87 | 0.231280795 | 1.949632025 |
| M680T448_1 | Campesteryl linoleate | 680.4718 | C46H78O2 | 0.000399979 | 3.4 | 0.033621224 | 1.156449168 |
| M680T448_2 | Goyaglycoside c | 680.4845 | C38H62O9 | 6.22653E-05 | 4.21 | 0.011106006 | 1.746868746 |
| M687T443 | DG(24_1(15Z)_18_2(9Z,12Z)_0_0) | 686.5938 | C45H82O5 | 0.014936787 | 1.83 | 0.239021275 | 1.5129878 |
| M767T422_1 | PC(P-16_0_18_1(11Z)) | 766.5253 | C42H82NO7P | 0.044502749 | 1.35 | 0.361817412 | 1.194332277 |
| M783T442_2 | PC(16_0_18_1(11Z)) | 782.5618 | C42H82NO8P | 0.00535851 | 2.27 | 0.150845328 | 2.430275063 |
| M843T379_2 | PC(16_1(9Z)_24_1(15Z)) | 842.6713 | C48H92NO8P | 0.006208102 | 2.21 | 0.164562503 | 2.234279448 |
| M845T443 | TG(14_0_16_0_22_1(13Z)) | 844.7675 | C55H104O6 | 0.004433819 | 2.35 | 0.139161019 | 1.604679377 |
| M116T48 | Aspartate semialdehyde | 116.0351 | C4H7NO3 | 0.04454531 | 1.35 | 0.236296643 | 1.98401617 |
| M117T55_1 | 2-Hydroxy-3-methylbutyric acid | 117.0555 | C5H10O3 | 0.028337292 | 1.55 | 0.191545743 | 2.184823962 |
| M119T53 | Propionic acid | 119.0348 | C3H6O2 | 0.007165369 | 2.14 | 0.104651865 | 1.5430868 |
| M121T56 | Benzoic acid | 121.0657 | C7H6O2 | 0.030958448 | 1.51 | 0.197854122 | 1.383470558 |
| M125T53 | 2-Hydroxyethanesulfonate | 125.0242 | C2H6O4S | 0.006924759 | 2.16 | 0.10400122 | 1.399648672 |
| M129T63 | chloric acid | 128.9594 | ClHO3 | 0.038405048 | 1.42 | 0.219209812 | 1.574449689 |
| M129T76 | (R)-2,3-Dihydroxy-3-methylvalerate | 129.0551 | C6H12O4 | 0.001288701 | 2.89 | 0.050874438 | 1.29812574 |
| M131T51_1 | Asparagine | 131.046 | C4H8N2O3 | 0.030133715 | 1.52 | 0.196145009 | 1.106976186 |
| M133T44 | DL-Malic acid | 133.0141 | C4H6O5 | 0.003601271 | 2.44 | 0.078497367 | 2.808590491 |
| M135T56 | Hypoxanthine | 135.0311 | C5H4N4O | 0.003009036 | 2.52 | 0.071947406 | 2.637724184 |
| M149T53 | D-Apiose | 149.045 | C5H10O5 | 0.015486532 | 1.81 | 0.146527155 | 1.531749873 |
| M151T55 | Xanthine | 151.0399 | C5H4N4O2 | 0.00322098 | 2.49 | 0.074423229 | 1.53833523 |
| M155T51 | Catechol | 155.0349 | C6H6O2 | 0.009118377 | 2.04 | 0.11717358 | 1.806257323 |
| M167T50 | 3-Mercaptolactic acid | 167.0018 | C3H6O3S | 0.009528182 | 2.02 | 0.119748729 | 1.003429068 |
| M172T74 | N-Acetylleucine | 172.0977 | C8H15NO3 | 0.003905196 | 2.41 | 0.079526997 | 1.214633791 |
| M173T48 | 5,6-Dihydrothymine | 173.0566 | C5H8N2O2 | 0.015846857 | 1.8 | 0.148360555 | 1.47650509 |
| M178T75 | Hippuric acid | 178.0508 | C9H9NO3 | 0.011533576 | 1.94 | 0.13172711 | 1.530649017 |
| M182T77 | Tyramine | 182.0821 | C8H11NO | 0.000383798 | 3.42 | 0.03180231 | 2.118296466 |
| M187T52 | FA 8_1 | 187.0958 | C8H14O2 | 0.005440261 | 2.26 | 0.092063008 | 1.213359439 |
| M187T54 | Azelaic acid | 187.1086 | C9H16O4 | 0.025810949 | 1.59 | 0.184527093 | 2.144693086 |
| M188T44 | AminoDHQ | 188.0561 | C7H11NO5 | 0.002549735 | 2.59 | 0.064836109 | 1.336236388 |
| M193T48 | D-Glucuronate | 193.0353 | C6H10O7 | 0.000684259 | 3.16 | 0.038635797 | 1.647030962 |
| M196T54 | L-Dopa | 196.0612 | C9H11NO4 | 0.0050985 | 2.29 | 0.089756017 | 1.935044833 |
| M197T49 | Mercaptopurine | 197.0125 | C5H4N4S | 0.045071148 | 1.35 | 0.237234758 | 1.191432164 |
| M197T73 | Geranial | 197.1183 | C10H16O | 0.019760505 | 1.7 | 0.168393585 | 1.685168406 |
| M199T233 | beta-Geraniol | 199.1338 | C10H18O | 0.03377875 | 1.47 | 0.206540296 | 1.007490001 |
| M199T80 | 3-methyl-4-cis-hydroxy-2-butenal | 199.0972 | C5H8O2 | 0.006510843 | 2.19 | 0.100939063 | 1.915453964 |
| M203T110 | Propyl acetate | 203.1282 | C5H10O2 | 8.02243E-05 | 4.1 | 0.017037907 | 2.091491834 |
| M203T198 | 3-Methylcatechol 1-sulfate | 203.1284 | C7H8O5S | 0.00163936 | 2.79 | 0.055579625 | 1.108534768 |
| M203T51 | Alpha-ketobutyrate | 203.056 | C4H6O3 | 0.029701202 | 1.53 | 0.196145009 | 1.168135807 |
| M211T226 | Dodecanedioic acid | 211.1339 | C12H22O4 | 0.020442434 | 1.69 | 0.170349548 | 1.173814782 |
| M211T50 | 5-Carboxyvanillate | 211.028 | C9H8O6 | 0.02688034 | 1.57 | 0.18581574 | 1.634974564 |
| M217T71_1 | 4-hydroxy-2-nonenal (4-HNE) | 217.1077 | C9H16O2 | 1.93692E-05 | 4.71 | 0.008462567 | 2.771262197 |
| M217T95 | (+)-Neomenthol | 217.1443 | C10H20O | 0.000595324 | 3.23 | 0.036596351 | 1.246382833 |
| M219T242 | Menadiol | 219.0694 | C11H10O2 | 0.04640032 | 1.33 | 0.238248576 | 2.094698156 |
| M223T49 | 1,2-Dihydroxy-3,4-epoxy-1,2,3,4-tetrahydronaphthalene | 223.0643 | C10H10O3 | 0.000117694 | 3.93 | 0.01885461 | 2.130111363 |
| M225T55_1 | Eugenol | 225.0767 | C10H12O2 | 0.024095832 | 1.62 | 0.18179681 | 2.050266455 |
| M227T54_2 | 2,4-diacetamido-2,4,6-trideoxy-beta-L-gulopyranose | 227.1035 | C10H18N2O5 | 0.004946224 | 2.31 | 0.0877179 | 1.65721838 |
| M227T72_1 | 5-Oxo-1,2-campholide | 227.0924 | C10H14O3 | 0.026559789 | 1.58 | 0.18581574 | 2.053876076 |
| M229T72 | Diazoxide | 229.1439 | C8H7ClN2O2S | 1.49353E-06 | 5.83 | 0.001788452 | 2.528820564 |
| M229T89 | Valylisoleucine | 229.1553 | C11H22N2O3 | 0.000211641 | 3.67 | 0.025063012 | 2.029860013 |
| M233T396 | Germacrene A acid | 233.1544 | C15H22O2 | 0.013537208 | 1.87 | 0.140690991 | 1.027047512 |
| M235T204 | 1,2-Dihydroxy-7-hydroxymethylnaphthalene | 235.0644 | C11H10O3 | 0.003067441 | 2.51 | 0.072158857 | 1.488808864 |
| M235T95 | 3-(4-methylthio)butylmalate | 235.0641 | C9H16O5S | 0.00136357 | 2.87 | 0.05160092 | 1.9965768 |
| M240T212 | Falcarinolone | 240.1241 | C17H22O2 | 0.045260107 | 1.34 | 0.237234758 | 1.546490408 |
| M240T87 | 6-Phosphonoglucono-D-lactone | 240.1238 | C6H11O9P | 0.045906851 | 1.34 | 0.237234758 | 1.432830802 |
| M241T45 | (E)-3-(2,3-Dihydroxyphenyl)-2-propenoic acid | 241.0383 | C9H8O4 | 0.000430479 | 3.37 | 0.032839435 | 1.869683067 |
| M243T142 | 6-(1-hydroxy-3-oxobutyl)-7-methoxy-2H-chromen-2-one | 243.1235 | C14H14O5 | 0.001266439 | 2.9 | 0.050874438 | 1.506596334 |
| M247T73 | 2,2'_5',2'-Terthiophene | 247.115 | C12H8S3 | 0.001013987 | 2.99 | 0.04321925 | 2.18063053 |
| M248T57 | N-(3-Methylbut-2-EN-1-YL)-9H-purin-6-amine | 248.0531 | C10H13N5 | 0.032608969 | 1.49 | 0.203003503 | 1.372146176 |
| M251T219 | 5-Hydroxymethyl-2-furancarboxaldehyde | 251.0593 | C6H6O3 | 0.027059535 | 1.57 | 0.186582674 | 1.137528264 |
| M255T288 | Nicotinic acid ribonucleoside | 255.0721 | C11H14NO6+ | 0.000410006 | 3.39 | 0.03226045 | 2.422693985 |
| M255T348 | Demethylmedicarpin | 255.2337 | C15H12O4 | 0.005401284 | 2.27 | 0.091978745 | 1.633220665 |
| M255T44_1 | Norbaeocystin | 255.0542 | C10H13N2O4P | 0.003200325 | 2.49 | 0.074303198 | 1.05211578 |
| M259T54 | Arginine, N2-benzoyl | 259.1186 | C13H18N4O3 | 0.00987358 | 2.01 | 0.121650463 | 2.132982852 |
| M263T197 | 1,7-Diphenyl-4-hepten-3-one | 263.1397 | C19H20O | 0.011469092 | 1.94 | 0.13172711 | 1.042120426 |
| M264T43 | Pseudobaptigenin | 264.018 | C16H10O5 | 0.00741811 | 2.13 | 0.106105468 | 2.344532521 |
| M265T323 | Juvenile hormone III | 265.1474 | C16H26O3 | 0.015066652 | 1.82 | 0.146283498 | 1.212000123 |
| M266T49 | (-)-Maackiain | 266.0337 | C16H12O5 | 0.020540297 | 1.69 | 0.170349548 | 1.055793259 |
| M267T324 | alpha-eudesmol | 267.1961 | C15H26O | 0.034097707 | 1.47 | 0.20717267 | 1.301143589 |
| M267T354 | Drimenol | 267.1962 | C15H26O | 0.005643632 | 2.25 | 0.094178114 | 2.038471574 |
| M269T331 | Emodin | 269.2118 | C15H10O5 | 0.000177044 | 3.75 | 0.023635417 | 2.243969506 |
| M269T387 | 16-oxo-palmitate | 269.2122 | C16H30O3 | 0.000244282 | 3.61 | 0.025150844 | 1.744642545 |
| M269T413 | Apigenin | 269.2122 | C15H10O5 | 0.026048738 | 1.58 | 0.184638086 | 1.055520882 |
| M270T58 | (R)-N-(Quinuclidin-3-yl)furo(2,3-C)pyridine-5-carboxamide | 270.0359 | C15H17N3O2 | 0.000781826 | 3.11 | 0.038710973 | 1.842566546 |
| M273T102 | Ptaerochromenol | 272.9971 | C15H14O5 | 0.000789362 | 3.1 | 0.038710973 | 1.894288178 |
| M275T233 | CAI-1 | 275.1858 | C13H26O2 | 0.005688729 | 2.24 | 0.094532846 | 1.780009478 |
| M275T419 | 9-HOTrE | 275.2011 | C18H30O3 | 0.023984687 | 1.62 | 0.181528198 | 1.648992364 |
| M281T443 | 2-hydroxystearate | 281.2453 | C18H36O3 | 0.046679515 | 1.33 | 0.238540202 | 1.41551958 |
| M285T245 | Cocaine | 285.1129 | C17H21NO4 | 0.004697054 | 2.33 | 0.085825963 | 2.032025514 |
| M285T267 | Hexadecanedioic acid | 285.2066 | C16H30O4 | 0.001519265 | 2.82 | 0.054899141 | 2.141869176 |
| M288T48 | Nebramine | 288.1088 | C12H26N4O5 | 0.002644134 | 2.58 | 0.0656891 | 1.370192737 |
| M293T185 | Nivalenol | 293.1002 | C15H20O7 | 0.006219354 | 2.21 | 0.099382135 | 2.0177702 |
| M293T301 | 8(R)-Hydroperoxylinoleic acid | 293.2118 | C18H32O4 | 0.006662913 | 2.18 | 0.101657018 | 1.354588618 |
| M297T88_2 | Artemether | 297.1706 | C16H26O5 | 0.018294756 | 1.74 | 0.158995657 | 2.092580232 |
| M299T72_3 | Farrerol | 299.1613 | C17H16O5 | 0.020835939 | 1.68 | 0.171175254 | 1.686010268 |
| M307T374 | warfarin | 307.2641 | C19H16O4 | 0.00054065 | 3.27 | 0.036555062 | 1.678813779 |
| M309T100 | trans-cinnamoyl-beta-D-glucoside | 309.1005 | C15H18O7 | 0.035505704 | 1.45 | 0.211450326 | 1.7784833 |
| M309T449 | 11Z-Eicosenoic acid（20_1） | 309.2793 | C20H38O2 | 0.001468503 | 2.83 | 0.053694534 | 2.019603973 |
| M311T335 | methyl gibberellin A9 | 311.1681 | C20H26O4 | 0.001242238 | 2.91 | 0.050594875 | 1.894404685 |
| M317T384 | Sventenic acid | 317.2116 | C20H30O3 | 0.001703219 | 2.77 | 0.056202153 | 2.340725419 |
| M319T232 | D-Alanyl-D-alanine | 319.1661 | C6H12N2O3 | 0.002543344 | 2.59 | 0.064836109 | 2.330677684 |
| M319T403 | FA 20_3;O2 | 319.2278 | C20H34O4 | 0.000799125 | 3.1 | 0.038793889 | 1.995479459 |
| M325T329 | FA 18_2 | 325.2379 | C18H32O2 | 0.01131379 | 1.95 | 0.131338344 | 1.773930515 |
| M327T446 | Docosahexaenoic acid | 327.2328 | C22H32O2 | 0.00458251 | 2.34 | 0.085362564 | 2.241291805 |
| M327T55 | S-Methyl-L-methionine | 327.1448 | C6H14NO2S+ | 0.003883705 | 2.41 | 0.079526997 | 1.269220668 |
| M329T104 | (R)-2-Hydroxy-2H-1,4-benzoxazin-3(4H)-one | 329.0809 | C8H7NO3 | 0.002035929 | 2.69 | 0.059662662 | 1.345055232 |
| M341T53 | 2-O-beta-D-mannopyranosyl-D-mannopyranose | 341.1456 | C12H22O11 | 0.033904451 | 1.47 | 0.206782729 | 1.108997227 |
| M345T321 | 11-cis-Retinaldehyde | 345.2097 | C20H28O | 0.018226836 | 1.74 | 0.158876059 | 1.593804629 |
| M347T223 | Chelerythrine | 347.1169 | C21H18NO4+ | 0.036970635 | 1.43 | 0.214331569 | 1.682498752 |
| M347T360 | Testololactone | 347.1894 | C19H26O3 | 0.007357364 | 2.13 | 0.105866739 | 1.851933547 |
| M351T43 | Sedoheptulose 7-phosphate | 351.0385 | C7H15O10P | 0.030708093 | 1.51 | 0.19724091 | 1.013329509 |
| M351T50 | Moschamine | 351.1402 | C20H20N2O4 | 0.02058104 | 1.69 | 0.170349548 | 1.935339178 |
| M353T418 | Neochlorogenic acid | 353.3421 | C16H18O9 | 0.00478112 | 2.32 | 0.086265729 | 2.011327402 |
| M353T437 | 22-oxodocosanoic acid | 353.3055 | C22H42O3 | 0.016532135 | 1.78 | 0.151354181 | 1.652022972 |
| M361T271 | 18-Hydroxycorticosterone | 361.204 | C21H30O5 | 0.022369993 | 1.65 | 0.175201522 | 1.467689816 |
| M362T265 | Tryprostatin A | 362.1883 | C22H27N3O3 | 0.003959497 | 2.4 | 0.079620678 | 2.141845774 |
| M367T414 | Lignoceric acid (C24) | 367.3571 | C24H48O2 | 0.00135091 | 2.87 | 0.05160092 | 1.417951244 |
| M373T203 | Kievitone hydrate | 373.129 | C20H22O7 | 0.023275045 | 1.63 | 0.179262609 | 2.180052531 |
| M381T83 | S-Adenosylmethionine | 381.112 | C15H23N6O5S+ | 0.030624832 | 1.51 | 0.19724091 | 1.81716802 |
| M385T285 | ST 24_4;O4 | 385.2373 | C24H34O4 | 0.019114327 | 1.72 | 0.16551974 | 1.39157033 |
| M393T262_2 | Tylactone | 393.2637 | C23H38O5 | 0.011539141 | 1.94 | 0.13172711 | 2.10335308 |
| M439T252 | Rotenone | 439.1454 | C23H22O6 | 0.044711056 | 1.35 | 0.236549278 | 1.241524387 |
| M447T443 | 27-Hydroxycholesterol | 447.3468 | C27H46O2 | 0.002269475 | 2.64 | 0.061801329 | 2.038204011 |
| M449T319 | Copalyl diphosphate | 449.3259 | C20H36O7P2 | 0.0470969 | 1.33 | 0.239521376 | 1.771399158 |
| M453T267_2 | 1-palmitoyl-dihydroxyacetone-phosphate | 453.2838 | C19H37O7P | 0.012028448 | 1.92 | 0.135067109 | 2.142738719 |
| M455T346_3 | gamma-Tocotrienol | 455.3173 | C28H42O2 | 0.01609826 | 1.79 | 0.149055612 | 1.684477223 |
| M462T336_2 | Micronomicin | 462.2988 | C20H41N5O7 | 0.012489278 | 1.9 | 0.137392498 | 1.084052321 |
| M481T377_2 | beta-Apo-4-carotenal | 481.3558 | C35H46O | 0.002096763 | 2.68 | 0.060658902 | 2.029929932 |
| M493T418 | Retosiban | 493.3483 | C27H34N4O5 | 0.000648111 | 3.19 | 0.038454599 | 1.955861097 |
| M497T335 | Artonol D | 497.3472 | C30H26O7 | 0.041827169 | 1.38 | 0.229475955 | 1.223532118 |
| M499T250 | Ubiquinone-1 | 499.3082 | C14H18O4 | 0.00632741 | 2.2 | 0.099382135 | 1.730097963 |
| M505T366_2 | Theasapogenol A | 505.3512 | C30H50O6 | 0.028743569 | 1.54 | 0.193522411 | 1.515142238 |
| M507T286 | 2alpha-(3-Hydroxypropyl)-1alpha,25-dihydroxy-19-norvitamin D3 | 507.2948 | C29H50O4 | 0.035137872 | 1.45 | 0.210040563 | 1.44241816 |
| M509T348 | ecdysone | 509.3454 | C27H44O6 | 0.017151738 | 1.77 | 0.15465526 | 1.652877106 |
| M529T276_1 | Physapubescin | 529.2698 | C30H42O8 | 0.001917205 | 2.72 | 0.057301163 | 1.621624414 |
| M555T261 | Dronedarone | 555.2989 | C31H44N2O5S | 0.045694126 | 1.34 | 0.237234758 | 1.416665035 |
| M559T407 | 10,11-epoxy-3,11-dimethyl-7-ethyl-2,6-tridecadienoic acid | 559.3991 | C17H28O3 | 0.004481948 | 2.35 | 0.085139306 | 1.455031655 |
| M565T237_1 | Dolichol diphosphate | 565.2712 | C25H46O7P2 | 0.045223656 | 1.34 | 0.237234758 | 1.226635291 |
| M578T350 | Astaxanthin | 578.349 | C40H52O4 | 0.017730259 | 1.75 | 0.156706387 | 2.094672741 |
| M596T327 | Neomycin | 596.2903 | C23H46N6O13 | 0.020129166 | 1.7 | 0.170018934 | 2.367249385 |
| M721T262_2 | Octaprenyl diphosphate | 721.436 | C40H68O7P2 | 0.000579067 | 3.24 | 0.036596351 | 1.386497626 |
| M748T426_2 | PG 34_1 | 747.5252 | C40H77O10P | 0.013192645 | 1.88 | 0.140690991 | 1.800784133 |
| M871T386_2 | (2-Hydroxy-3-phosphonooxypropyl) octadec-9-enoate | 871.4852 | C21H41O7P | 0.010228103 | 1.99 | 0.123508199 | 1.1706426 |
